# Epigenetic modifications are associated with mRNA and cytokine expression changes in chronic rhinosinusitis: a multiomics study from the United States

**DOI:** 10.3389/falgy.2025.1606255

**Published:** 2025-06-05

**Authors:** Devyani Lal, Tripti Brar, Chantal McCabe, Erik Jessen, Nitish Kumar, Pedro Lança Gomes, Michael J. Marino, Amar Miglani, Hirohito Kita

**Affiliations:** ^1^ Department of Otolaryngology-Head & Neck Surgery, Mayo Clinic in Arizona, Phoenix, AZ, United States; ^2^ Department of Quantitative Health Sciences, Mayo Clinic, Rochester, MN, United States; ^3^ Department of Allergy, Asthma and Clinical Immunology, Mayo Clinic, Scottsdale, AZ, United States

**Keywords:** epigenetics, chronic rhinosinusitis, transcriptomics, proteomics, multiomics, cytokines, differentially methylated DNA, differentially expressed mRNA

## Abstract

**Objectives/hypothesis:**

Chronic rhinosinusitis (CRS) may be triggered by environmental insults. We hypothesized that CRS results from epigenetic modifications of host DNA from external insults, leading to downstream RNA/DNA gene expression changes and immuno-mechanical disruptions. We therefore performed a multi-omics study integrating epigenetic (DNA methylation), transcriptomic (mRNA), and proteomic (cytokine) data of CRS sinonasal tissue to visualize interactions amongst these modalities to study our hypothesis.

**Methods:**

Sinonasal tissue was collected from 14 prospectively enrolled CRS and control subjects. Cytokine, mRNA transcriptome, and DNA methylome analysis were performed. Multi-omics analysis via joint dimensional reduction (JDR) was conducted.

**Results:**

Multi-omics unsupervised clustering separated subjects into two distinct groups: one cluster of 9 CRS subjects and another with 3 controls and 2 non-eosinophilic CRSsNP subjects. DNA methylation, followed by mRNA expression, contributed most to cluster assignment. DNA methylation was the most significant data modality contributing to total variance on JDR. Cytokines critical in CRS (IL-5, IL-13, IL-10, IFN*γ*, IL-6) associated with hundreds of differentially methylated regions (DMRs) and mRNA. On conjoint analyses, common upstream DMRs and mRNAs were linked to cytokines IL-5 and IL-13, cytokines IL-10 and IFN*γ*, and cytokines IFN*γ* and IL-6, respectively.

**Conclusions:**

Our results support the hypothesis that environmental insults may be significant drivers of CRS pathogenesis through epigenetic mechanisms that result in dysregulated mRNA transcription and cytokine expression. The most novel part of this study is our multi-omics approach that used integration of epigenetic (DNA methylation), transcriptomic (mRNA), and proteomic (cytokine) data to uncover insights into CRS pathogenesis; this is the first of its kind in CRS etiopathogenesis. The multi-omics analysis clearly separated clusters of control and CRS subjects, demonstrating its validity in future research. The study also identified interactions of methylated DNA, mRNA, and cytokines in CRS pathogenesis, highlighting novel molecules and pathways that may be potential therapeutic targets.

## 1 Introduction

A complex interaction of unfavorable environmental insults in the susceptible host has been postulated to disrupt normal homeostatic mechanisms in chronic rhinosinusitis (CRS) (1). Proposed external stressors (“the environment”) include microbial pathogens, microbiome dysbiosis, exposure to allergens, and air pollution (1). In addition, genetic susceptibility may be one of several host factors that result in disease. Even though familial clustering has long been reported, it is unclear whether familial clustering results from shared genes or shared environments, as identifiable monogenic alterations have not been identified in most CRS patients (2–5). In a large population-based study from Utah, U.S.A., 1,638 CRS with nasal polyposis (CRSwNP) and 24,200 CRS sans NP (CRSsNP) subjects were matched to random controls; 1st and 2nd-degree relatives were found to have a 4.1-fold and 3.3-fold elevated risk for CRSwNP, respectively. For CRSsNP, 1st and 2nd-degree relatives had a 2.4-fold and 1.4-fold risk, respectively (2). Interestingly, spouses of CRSsNP patients were also found to have a 2-fold increased risk of CRSsNP (2). In Sweden, Bohman, et al. (6) found that the prevalence of CRSwNP in relatives was 13.4% vs. 2.7% in controls; a relative risk of 4.9 in the first-degree relatives. These studies generate questions about the pathogenic roles of genes, shared environments, or both.

Epigenetics is the study of environmental influences on gene expression. Epigenetic studies are particularly helpful in disease states such as CRS, where multiple host or environmental factors may influence disease pathogenesis (7). External impact is modulated through mechanisms such as DNA methylation, histone modifications, non-coding RNAs, and alternative polyadenylation (APA) (8, 9). Epigenetic changes can notably persist and be passed to the progeny for 2–3 generations. Epigenetics may help explain both familial clustering and the increase in prevalence. Early studies on CRS epigenetics have shown several differences in DNA methylation between CRS and control tissue (10, 11). However, most of these are based in Asia, with only two of our previous studies being conducted in the United States. In this current study, our goal was to investigate the association, if any, of epigenetic modifications and mRNA transcriptomic and proteomic changes characterizing CRS. Transcriptomics analyzes RNA molecules, such as messenger RNA (mRNA), to understand gene expression (4, 12, 13) and has helped identify mechanistic pathways. However, most CRS transcriptomics studies have been conducted in Asia (14–17), with three studies incorporating non-Asian subjects (18–20) this is a relatively novel approach in North America, where population genetics and the environment differ (21, 22). When connecting transcriptomics with proteomics, studies have been divergent, reporting a correlation between CRS mRNA expression (transcriptome) and the proteome (17, 23, 24), as well as discordance (21) highlighting the need for further research using a multi-omics approach. Multiomics studies incorporate multiple modalities with large data sets through bioinformatics tools to help uncover complex relationships and interactions of biological processes at various levels, and can also help identify pathogenetic pathways that may not be apparent when studying each “omics” field individually. However, multiomics analyses involving the epigenome, transcriptome, and proteome (cytokine) have previously not been studied in CRS.

We hypothesize that external insults cause epigenetic modifications of host DNA, resulting in unfavorable DNA and associated RNA and protein expressions, which result in immuno-mechanical disruptions associated with CRS pathogenesis. We tested our hypothesis by performing multi-omics analyses integrating epigenetic (DNA methylation), transcriptomic (mRNA), and proteomic (cytokine) data of CRS sinonasal tissue. Our secondary goal was to visualize interactions amongst these modalities to uncover novel molecules and pathways with potential roles in CRS pathogenesis.

## 2 Methods

This study was conducted at a tertiary-level hospital in Arizona after approval from the institutional review board (IRB ID: 16-008609). Subjects were classified into controls and CRS based on nasal endoscopy and sinus CT according to 2015 consensus guidelines from the American Academy of Otolaryngology-Head and Neck Surgery (25). CRS subjects were further classified into CRSwNP and CRSsNP. Patients on systemic corticosteroids, biological therapy, and systemic or topical antibiotics in the last 4 weeks were excluded, so as not to affect the baseline cytokine profile of sinonasal tissue. Control subjects were undergoing transsphenoidal endoscopic resection of pituitary adenoma and were negative on CT and endoscopy for sinusitis and had no nasal history suggestive of allergic rhinitis. Prospective data was collected on demographics, clinical diagnoses, and disease severity [patient reported 22-item sinonasal outcome test (SNOT-22) scores (26) and Lund Mackay (27) Sinus CT scores].

STATA BE/18.0 was used to assess any differences in age and sex distribution between the cohorts. The Mann–Whitney *U*-test was used to compare the difference in age distribution, and Fisher's exact test was used to compare the gender distribution between the cohorts. A *p*-value of <0.05 was chosen as the criterion of statistical significance. Sinonasal tissue samples for all multi-omics analyses were obtained at a single time point under direct endoscopic guidance for 11 CRS and 3 control subjects. Since sinonasal mucosal samples were obtained during surgery, standard surgical aseptic precautions were used. Specimens were stored at −80°C until analysis. Samples were placed into sterile 7 ml polycarbonate tubes (Sarstedt 71.9923.610) and frozen within 15 min in a −90°C bath of Novec-engineered fluid (3M HFE-7000) cooled in a HistoChill freezing bath (SP Scientific HC80A0). Ethmoidal tissue was used for DNA methylation and cytokine studies. RNA sequencing was performed on ethmoidal tissue in CRS patients and inferior turbinate tissue in controls per IRB approval. A part of the ethmoidal tissue was sent in formalin at the time of surgery for structured histopathology analysis as described by Snidvongs et al. (28) Subjects with tissue eosinophils ≥10 eos/hpf were classified as eosinophilic CRS (eCRS) and those with <10 eos/hpf as non-eosinophilic CRS (neCRS).

### 2.1 DNA methylation

DNA extraction was done using the QIAamp DNA Mini kit by Qiagen (Reference no. 51306). Reduced Representation Bisulfite Sequencing (RRBS) Library prep and Sequencing were done on Illumina's HiSeq4000. RRBS data were analyzed using a streamlined analysis and annotation pipeline (SAAP) for RRBS, SAAP-RRBS (29). Cytosine followed by a guanine nucleotide (CpG) loci were called differentially methylated CpGs (DMCs) when *p* ≤ 0.05 and the mean methylation difference for the CpG loci between groups was at least 5% (delta ≥5%). A requirement of having at least four CpG loci within a candidate differentially methylated region (DMR) was set. Further details are included in the Supplementary Section.

### 2.2 RNA-Sequencing

RNA samples underwent library prep using Illumina TruSeq® RNA Exome Library Prep kit (San Diego, CA). Libraries were sequenced in 2 pools per lane on an Illumina HiSeq 4,000 (100 × 2 paired-end reads) and base-calling using Illumina's RTA v2.7.7. Paired-end RNA sequencing reads were processed through the RNA-Seq bioinformatics pipeline, MAP-RSeq v3.1.4 (30). Differentially expressed genes (DEG) were identified from raw gene counts using edgeR 2.6.2 (31). DEGs were reported with log2 fold change and False Discovery Rate (FDR <5%). Canonical pathway analysis using Ingenuity Pathway Analysis (IPA) software (Ingenuity® Systems) identified significant pathways (*p*-value <5%). Further details are included in the Supplementary Section.

### 2.3 Cytokine analysis

Frozen specimens were weighed, thawed, mixed with phosphate-buffered saline (PBS) and protease inhibitors (Millipore Sigma, Burlington, MA), and homogenized. Supernatants were collected after centrifugation. Cytokine and chemokine levels (48-plex) were measured using a Millipore multiplex kit (Billerica, MA) on a Bio-Rad MAGPIX multiplex reader (Hercules, CA). The concentrations of cytokines were normalized to the concentration of total protein in each sample. Total protein was analyzed by using a BCA Protein Assay Kit (Thermo Fisher Scientific). The values of cytokines were divided by the values of total protein. Samples below the minimum detectable concentration (MinDC) were assigned half the MinDC, and values above the standard curve limit were assigned the highest standard. Cytokines and chemokines detected in <10% of samples (17) were excluded. Eosinophil peroxidase (EPX) levels were assessed using an in-house sandwich enzyme-linked immunosorbent assay (ELISA), like that described by Ochkur et al. (32).

### 2.4 Multi-omics analysis

#### 2.4.1 Preprocessing

Batch effects were corrected using ComBat (33). Modality-specific normalization was followed by transformation and filtering to facilitate an equal contribution to the JDR model. Normalized-FPKM RNA-seq counts were log_10_ + 1e^−4^ transformed to achieve a Gaussian distribution. To balance feature counts across modalities, cytokines (with the fewest features) were filtered along with methylation and RNA features, which were significantly different between CRS and controls (FDR <5%).

#### 2.4.2 Modeling

Joint dimensionality reduction (JDR) was performed using the multi-omics factor analysis (MOFA) methodology to integrate data modalities and extract variability dimensions, called factors (34). The contribution of each modality to the variance explained by each factor was quantified. To determine the number of viable factors, a randomized dataset was used, and factors where this dataset contributed the most variation were disregarded. Default settings were used, with modifications to remove scaling between data modalities and to pre-scale the value ranges to ensure a more accurate comparison of feature loading weights between modalities.

#### 2.4.3 Analysis

Hierarchical all-against-all (HAllA) clustering was used to link quantitative and categorical clinical variables to factors, identifying features driving factor-sample distributions. Joint pathway analysis or kinase enrichment inference was used to analyze contributing modalities and features after identifying the associating factor to the clinical variable of interest.

## 3 Results

Table 1 details the clinical characteristics of the subjects. No statistically significant differences were found in the age and sex distributions between CRS cases and control subjects.

**Table 1:** Clinical characteristics of subjects.

Characteristic	CRS^a^ (*n* = 11) *N* (%) or Mean (SD^b^)	Controls (*n* = 3) *N* (%) or Mean (SD^b^)
Age	52.5 (13.0)	46.3 (20.8) *p* = 0.63
Gender (M/F)	2 (18.2%)/9 (81.8%)	2 (66.7%)/1 (33.3%) *p* = 0.17
Clinical diagnosis		Non-CRS (pituitary adenoma)
• CRSwNP^c^	3 (27.2%)	
• CRSsNP^d^	8 (72.7%)	
SNOT-22^e^	34.5 (31.0); missing in one subject	6 (2)
Lund-Mackay score	12.1 (3.9)	0.7 (0.6)
Previous sinus surgery	7 (73.7%)	0
Absolute serum eosinophils (×10^9^)	0.36 (0.15)	0.32 (0.13)
Tissue Eosinophil/HPF^f^		
• <10	5 (45.5%)	3 (100%)
• 10–100	4 (36.4%)	
• >100	2 (18.2%)	
Asthma		
• Positive	7 (73.7%)	0
• Negative	4 (36.3%)	3 (100%)
Allergic rhinitis		
• Positive	9 (81.9%)	0
• Negative	2 (18.1%)	1 (33.3%)
• Not evaluated	0	2 (66.7%)
AERD^g^		
• Possible	1 (9%)	0
• Negative	10 (91%)	3 (100%)
Smoking history		
• Yes	0 (18.1%)	1 (33.3%)
• No	8 (72.7%)	2 (66.7%)
• Unknown	3 (27.2%)	0
Current nasal steroid spray		
• Yes	2 (18.1%)	0
• No	5 (45.4%)	0
• Unknown	4 (36.3%)	0

^a^ CRS: Chronic rhinosinusitis.

^b^ SD: Standard deviation.

^c^ CRSwNP: CRS with nasal polyposis.

^d^ CRSsNP: CRS sans nasal polyposis.

^e^ SNOT-22: 22-item sinonasal outcome test.

^f^ HPF: high power field.

^g^ AERD: Aspirin Exacerbated Respiratory Disease.

### 3.1 The multi-omics approach was successful in separating CRS subjects from controls

Multi-omics unsupervised clustering separated CRS from Controls; DNA methylation modality most contributed to cluster assignment, followed by RNA transcripts.

**Figure 1:**
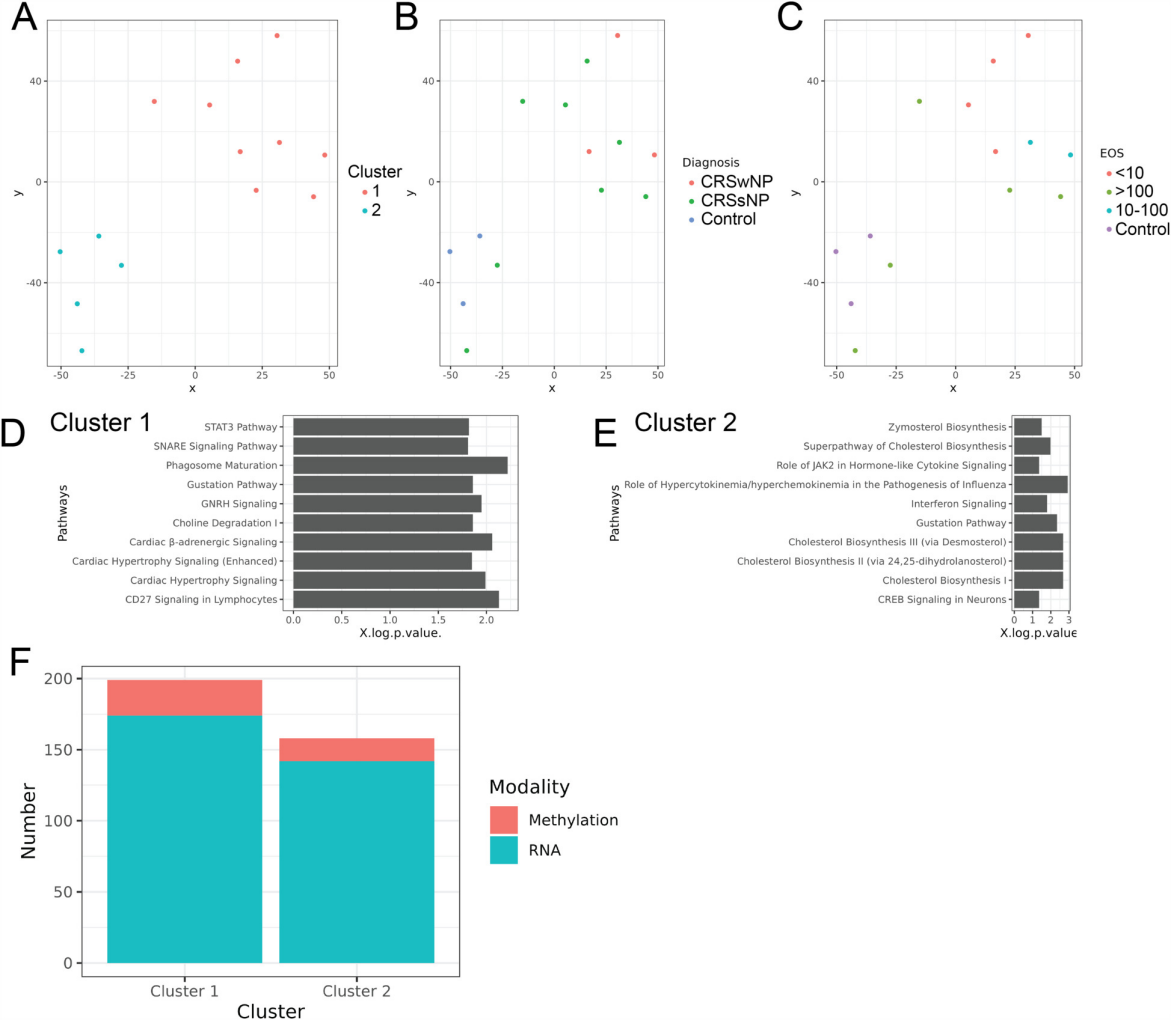
**(A)** Multiomics clustering of samples considering all three data modalities, **(B)** constituents of each cluster by diagnosis (control, CRSwNP, CRSsNP), **(C)** constituents of each cluster by tissue eosinophil count, **(D)** significant pathways associated with cluster 1 and **(E)** cluster 2, **(F)** number of features from each data modality contributing to cluster assignment.

Multi-omics unsupervised clustering revealed two distinct groups with clear separation (Figure 1A). Figure 1B depicts the clinical diagnosis of cluster constituents. Cluster 1 was found to be entirely constituted by CRS subjects (3 CRSwNP, 6 CRSsNP), and Cluster 2 included all 3 controls and 2 non-eosinophilic CRSsNP subjects. Figure 1C depicts cluster constituents based on tissue eosinophil status. Where all Cluster 2 constituents had <10 eos/hpf, 6 CRS subjects in Cluster 1 had high tissue eosinophilia (2 with >100 eos/hpf, and 4 with tissue eosinophils between 10 and 100 eos/hpf), and 2 had non-eosinophilic tissue. Next, we examined each cluster to identify associated pathways (Figure 1D, E). The known functions of the top pathways associated with Cluster 1 and Cluster 2 are depicted in Tables 2A, B, respectively. The modality that most contributed to cluster assignment was DNA methylation, followed by RNA transcripts (Figure 1F). DMRs and DE RNAs associated with both clusters were identified, the top 50 of which are listed in Tables 3A, B, respectively. Supplementary Tables S1A and S1B present the known functions of these genes.

**Table 2A:** Pathways associated with cluster 1 (9 CRS subjects).

Pathway	Considerations	Reference^a^
STAT3	^a^The protein encoded by STAT3 gene is a member of the STAT protein family. In response to cytokines and growth factors, STAT family members are phosphorylated by the receptor associated kinases, and then form homo- or heterodimers that translocate to the cell nucleus where they function as transcription activators. STAT3 protein is activated through phosphorylation in response to various cytokines and growth factors including IFNs, EGF, IL5, and IL6. This protein mediates expression of a variety of genes in response to cell stimuli, and thus plays a key role in many cellular processes such as cell growth and apoptosis. PIAS3 protein is a specific inhibitor of this protein. This gene also plays a role in regulating host response to viral and bacterial infections. Mutations in this gene are associated with infantile-onset multisystem autoimmune disease and hyper-immunoglobulin E syndrome. This gene participates in immune response or antiviral activity.	Liu 2021 (A1)
SNARE Signaling	SNARE proteins are critical in granule fusion events	Lacy 2011 (A2)
Phagosome maturation	Phagosome maturation is the process by which bacteria and other ingested particles are degraded. This pathway is regulated by p38 mitogen-activated protein kinase (MAPK), which is activated by toll-like receptors (TLRs).	Blander 2004 (A3)
Gustation	In CRSwNP, downregulated genes were enriched for gustatory sensory perception and tissue homeostasis,	Wang 2022 (A4)
GNRH signaling	Preprogonadotropin-releasing hormone-like protein (GnRH) is overexpressed in COVID-19 convalescent subjects	Huoman 2022 (A5)
Choline degradation I	Toll-like receptor (TLR) activation enhances choline uptake by macrophages through induction of choline transporter CTL1	Sanchez-Lopez 2019 (A6)
Cardiac B-adrenergic signaling	Reactive oxygen species formation regulates beta2-adrenergic receptor signal transduction	Michaeloudes 2022 (A7)
Cardiac hypertrophy signaling/enhanced signaling	Cellular metabolism, proliferation, non-coding RNAs, immune responses, translational regulation, and epigenetic modifications, positively or negatively regulate cardiac hypertrophy	Nakamura 2018 (A8)
CD27 Signaling in Lymphocytes	Is a costimulatory molecule of tumor necrosis factor receptor (TNFR) family, strongly expressed on activated CD4(+) and CD8(+) T lymphocytes	Behrendt 2010 (A9)

^a^ Sources: National Institutes of Health National Library of Medicine, https://www.ncbi.nlm.nih.gov/gene National Human Genome Research Institute https://www.genome.gov/genetics-glossary Last accessed August 16, 2024.

**Table 2B:** Pathways Associated with Cluster 2 (All 3 control and 2 CRS <10 eos/hpf subjects)

Pathway	Considerations	Reference
Zymosterol biosynthesis	Essential for membrane fluidity and function; important 2nd messenger lipids involved in developmental signaling	Germann 2005 (A10)
Superpathway of cholesterol biosynthesis; Cholesterol I, II (via 24,25-dihydrolanosterol); III (via desmosterol)	Cholesterol and cholesterol derivatives shape plasma membrane fluidity and lipid raft dynamics, affecting the formation of the immunological synapse and its downstream signalling events, modulating T-cell activation and function	Cardoso 2021 (A11)
Gustation pathway	In subjects diagnosed with CRSwNP, downregulated genes were predominantly enriched for gustatory sensory perception, tissue homeostasis, and muscle system process	Wang 2022 (A12)
CREB signaling in neurons	DNA-binding transcriptional regulator CREB is an intracellular protein that regulates expression of genes important in dopaminergic neurons	Wang 2018 (A13)
Role of JAK2 in hormone-like cytokine signaling	^a^Janus Kinase 2 (JAK2) protein has an N-terminal domain that is required for erythropoietin receptor association, an SH2 domain that binds STAT transcription factors, a pseudokinase domain and a C-terminal tyrosine kinase domain. Cytokine binding induces autophosphorylation and activation of this kinase. This kinase then recruits and phosphorylates signal transducer and activator of transcription (STAT) proteins. Growth factors like TGF-beta 1 also induce phosphorylation and activation of this kinase and translocation of downstream STAT proteins to the nucleus where they influence gene transcription. Mutations in this gene are associated with numerous inflammatory diseases and malignancies. This gene is a downstream target of the pleiotropic cytokine IL6 that is produced by B cells, T cells, dendritic cells, and macrophages to produce an immune response or inflammation. A nonsynonymous mutation in the pseudokinase domain of this gene disrupts the domains inhibitory effect and results in constitutive tyrosine phosphorylation activity and hypersensitivity to cytokine signalling. This gene and the IL6/JAK2/STAT3 signalling pathway is a therapeutic target for the treatment of excessive inflammatory responses to viral infections.	Wang 2016 (A14)
Role of hypercytokinemia/hyperchemokinemia in the pathogenesis of influenza	Hypercytokinemia/hyperchemokinemia precedes acute respiratory distress syndrome (ARDS) during influenza infection	Wei 2022 (A15)
Interferon signaling	Type I and II interferons and IL-27 inhibit ILC2 functions through the activation of STAT1	Duer 2016 (A16)

^a^ Sources: National Institutes of Health National Library of Medicine, https://www.ncbi.nlm.nih.gov/gene National Human Genome Research Institute https://www.genome.gov/genetics-glossary Last accessed August 16, 2024.

**Table 3A:** The Top 50 differentially methylated regions in DNA between cluster 1 and 2.

Gene name	Gene description	Gene type
C8orf31	Chromosome 8 open reading frame 31	Non-coding RNA
EDN2	Endothelin 2	protein-coding
MIR320E	MicroRNA 320e	Non-coding RNA
SP6	Sp6 transcription factor	protein-coding
NANS	N-acetylneuraminate synthase	protein-coding
COG1	Component of oligomeric Golgi complex 1	protein-coding
HEYL	HES related family bHLH transcription factor with YRPW motif like	protein-coding
ST3GAL4	ST3 beta-galactoside alpha-2,3-sialyltransferase 4	protein-coding
EXT1	Exostosin glycosyltransferase 1	protein-coding
SMAD3	SMAD family member 3	protein-coding
C1QTNF5	C1q and TNF related 5	protein-coding
MYCL	MYCL proto-oncogene, bHLH transcription factor	protein-coding
DNAJB6	DNA heat shock protein family (Hsp40) member B6	protein-coding
TULP1	TUB like protein 1	protein-coding
SLC44A2.2	Solute carrier family 44, member 2	protein-coding
SOX15	SRY-box transcription factor 15	protein-coding
SLC44A2	Solute carrier family 44, member 2	protein-coding
ZBTB16	Zinc finger and BTB domain containing 16	protein-coding
SEMA6C	semaphorin 6C	protein-coding
PEBP4	Phosphatidylethanolamine binding protein 4	protein-coding
EPHB3	EPH receptor B3	protein-coding
SPAG6	Sperm associated antigen 6	protein-coding
LINC00963	Long intergenic non-protein coding RNA 963	Non-coding RNA
SLC44A2.1	Solute carrier family 44, member 2	protein-coding
QRFP	Pyroglutamylated RFamide peptide	protein-coding
LINC00265	Long intergenic non-protein coding RNA 265	Non-coding RNA
SHISAL1	Shisa like 1	protein-coding
CACNA1H	Calcium voltage-gated channel subunit alpha1 H	protein-coding
PTPN21	Protein tyrosine phosphatase non-receptor type 21	protein-coding
GADD45B	Growth arrest and DNA damage inducible beta	protein-coding
FBLN1	Fibulin 1	protein-coding
ANKRD65	Ankyrin repeat domain 65	protein-coding
OSBPL5	Oxysterol binding protein like 5	protein-coding
KLK5	Kallikrein related peptidase 5	protein-coding
TFDP1	Transcription factor Dp-1	protein-coding
AGAP2-AS1	AGAP2 antisense RNA 1	Non-coding RNA
WFIKKN2	WAP, follistatin, immunoglobulin, kunitz, netrin domain containing 2	protein-coding
LINC01338	Long intergenic non-protein coding RNA 1338	Non-coding RNA
STMND1	Stathmin domain containing 1	protein-coding
SHANK2	SH3 and multiple ankyrin repeat domains 2	protein-coding
CD37	CD37 molecule	protein-coding
PRR25	Proline rich 25	protein-coding
SLURP2	Secreted LY6/PLAUR domain containing 2	protein-coding
CACNA1C-IT3	CACNA1C intronic transcript 3	Non-coding RNA
LINC01814	Long intergenic non-protein coding RNA 1814	Non-coding RNA
IL31RA	Interleukin 31 receptor A	protein-coding
FBLN7	Fibulin 7	protein-coding
FAM78A	Family with sequence similarity 78 member A	protein-coding
PRMT7	Protein arginine methyltransferase 7	protein-coding
PLA2G4C	Phospholipase A2 group IVC	protein-coding

Sources: National Institutes of Health National Library of Medicine, https://www.ncbi.nlm.nih.gov/gene National Human Genome Research Institute https://www.genome.gov/genetics-glossary Last accessed August 16, 2024.

**Table 3B:** The Top 50 Differentially Expressed mRNA between Clusters 1 and 2

Gene name	Gene description	Gene type
ATP2A3	ATP2A3 (ATPase Sarcoplasmic/Endoplasmic Reticulum Ca2+ Transporting 3) also known as SERCA3	Protein coding
PNCK	Pregnancy Up-Regulated Nonubiquitous CaM Kinase	Protein coding
CCDC88B	CCDC88B coiled-coil domain containing 88B [Homo sapiens (human)]	Protein coding
CST4	cystatin S	Protein coding
ARHGAP40	Rho GTPase activating protein 40	Protein coding
CST1	cystatin SN	Protein coding
LDLRAD2	low density lipoprotein receptor class A domain containing 2	Protein coding
PRB1	proline rich protein BstNI subfamily 1	Protein coding
CCDC183	coiled-coil domain containing 183	Protein coding
RILP	Rab interacting lysosomal protein	Protein coding
MAPK8IP3	mitogen-activated protein kinase 8 interacting protein 3	Protein Coding
PLXNB3	plexin B3	Protein Coding
SRPK3	SRSF protein kinase 3	Protein coding
ARHGF16	Rho guanine nucleotide exchange factor 16	Protein coding
CARD14	caspase recruitment domain family member 14	Protein coding
CHDH	choline dehydrogenase	Protein coding
JSRP1	junctional sarcoplasmic reticulum protein 1	Protein coding
PTPRH	protein tyrosine phosphatase receptor type H	Protein coding
ATP10B	ATPase phospholipid transporting 10B	Protein coding
FUT3	Also known as CD174/fucosyltransferase 3 (Lewis blood group)	Protein coding
EGLN3	egl-9 family hypoxia inducible factor 3	Protein coding
TMEM200A	transmembrane protein 200A	Protein coding
HBA2	hemoglobin subunit alpha 2	Protein coding
PRLR	prolactin receptor	Protein coding
LRFN5	leucine rich repeat and fibronectin type III domain containing 5	Protein coding
FRMD6	FERM domain containing 6	Protein coding
LUM	lumican	Protein coding
JAM2	junctional adhesion molecule 2	Protein coding
CNTN1	Contactin 1	Protein coding
SNHG14	Small nucleolar RNA host gene 14	Noncoding RNA
STAC	SH3 and cysteine rich domain	Protein coding
WNT5A	Wnt family member 5A	Protein coding
NELL2	Neural EGFL like 2	Protein coding
HMGN1P36	High mobility group nucleosome binding domain 1 pseudogene 36	Pseudogene
PTN	Pleiotrophin	Protein coding
SCN2B	Sodium voltage-gated channel beta subunit 2	Protein coding
AR	Androgen receptor	Protein coding
SNORD116-18	Small Nucleolar RNA, C/D Box 116-18	snoRNA
SNORD 116-25	Small Nucleolar RNA, C/D Box 116-25	snoRNA
TCP1	T-complex 1	Protein coding
SNORD62A	Small nucleolar RNA, C/D box 62A	snoRNA
SNORD62B	Small nucleolar RNA, C/D box 62B	snoRNA
GPC3	Glypican 3	Protein coding
CCDC36	Coiled-coil domain containing 36	Protein coding
CSMD3	CUB and Sushi multiple domains 3	Protein coding
SLIT2	Slit guidance ligand 2	Protein coding
ZNF660	Zinc finger protein 660	Protein coding
DPP6	Dipeptidyl peptidase like 6	Protein coding
NPNT	Nephronectin	Protein coding

snoRNA: Small nucleolar RNAs (snoRNAs), a class of small RNA molecules that primarily guide chemical modifications of other RNAs. Sources: National Institutes of Health National Library of Medicine, https://www.ncbi.nlm.nih.gov/gene National Human Genome Research Institute https://www.genome.gov/genetics-glossary Last accessed August 16, 2024.

### 3.2 DNA methylation was the most significant data modality contributing to total variance

**Figure 2:**
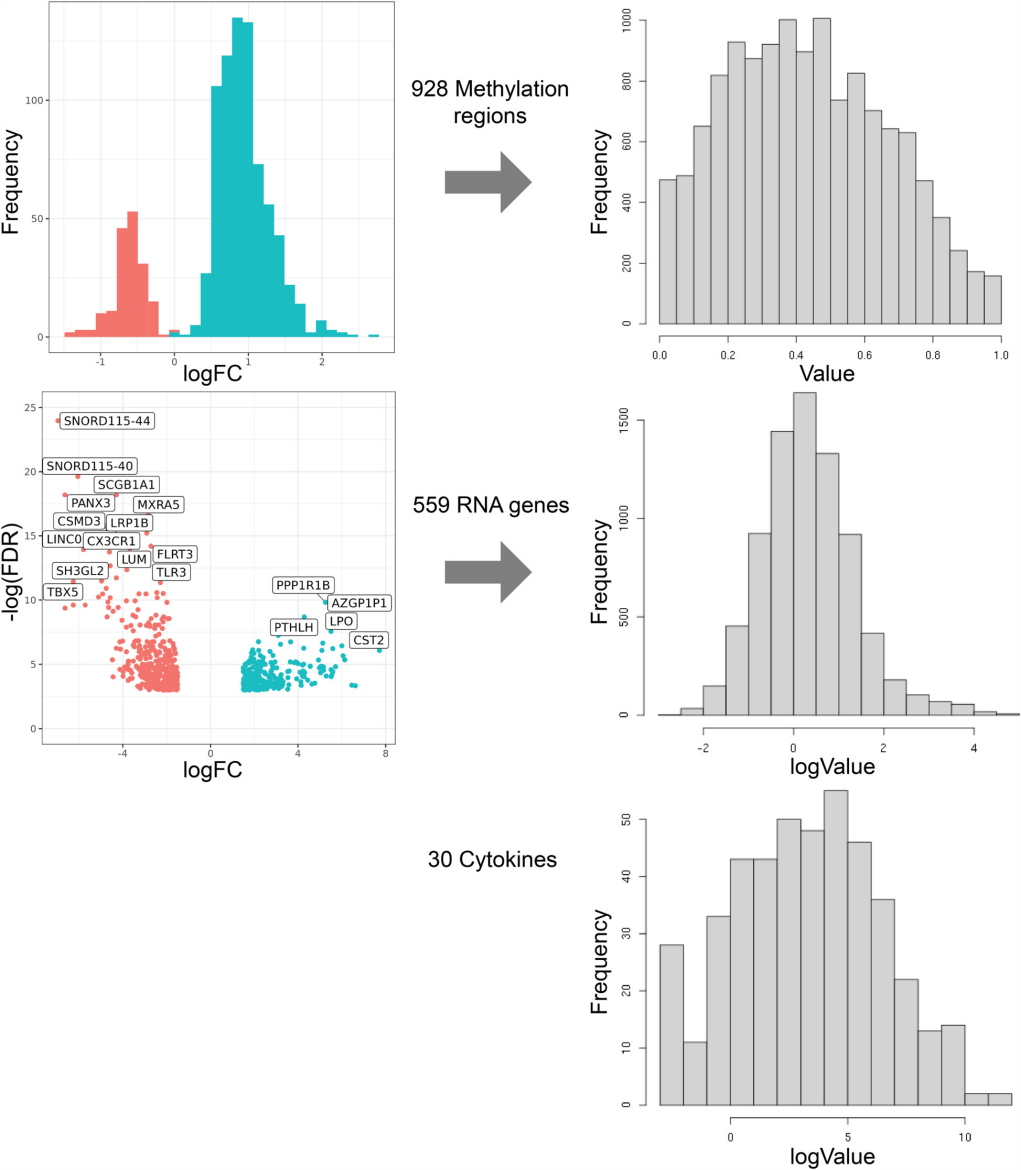
Filtering and transformation strategy for each data modality through volcano plots and histogram representations. Data value distributions were transformed to be as close to Gaussian as possible.

Tissue samples from 14 subjects demonstrated several unique features across DNA methylation, transcriptomic, and cytokine data. Figure 2 depicts the filtering and transformation strategy for each data modality. *DNA Methylation was the most significant data modality contributing to Total Variance.*

**Figure 3:**
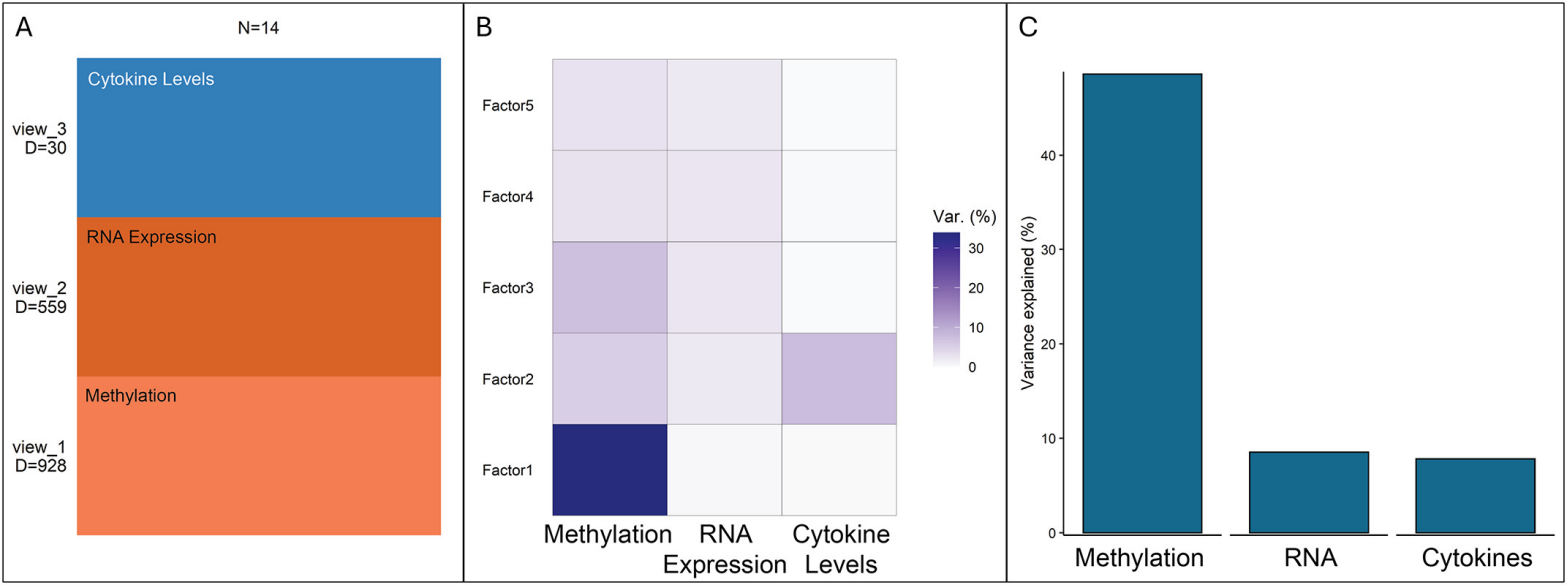
Total variance contribution from each data modality (methylation, RNA, cytokines) is shown: **(A)** total number of data points for each modality, **(B)** joint dimension reduction resulted in five dimensions of variation (“Factors”). The darker the color, the more the contribution to the individual factor from the modality (methylation, RNA, cytokine), **(C)** Modality contributing to the total variance.

JDR resulted in five dimensions of variation (“factors”), which captured the most significant patterns of information (Figure 3B). Methylation was the most significant data modality contributing to total variance (Figure 3C). Factor 1 is mostly driven by methylation. Factors 4 and 5 have similar contributions from methylation and RNA expression. Factor 3 is mostly correlated with methylation and less with RNA expression. Factor 2's important contribution comes from cytokines; however, it was also moderately correlated to DNA methylation, and weakly to RNA expression (Figure 3B). Table 4 details significant pathways associated with each factor as referenced by studies of inflammatory mechanisms.

**Table 4:** Significant pathways associated with each of the five factors shown in figure 2B.

Pathways—factor	Considerations	Reference
Pathways associated with Factor 1		
Transcriptional regulatory network in embryonic stem cells	Specifies gene expression and imparts distinct cellular phenotypes	Chan 2011 (A17)
Signaling by Rho family GTPases	Precise regulation of actin cytoskeletal dynamics as well as other immunological functions of leukocytes	Dipankar 2021 (A18)
Role of JAK1 and JAK3 in gamma-c cytokine signaling	Janus kinases are associated with intracellular domains of IL-2, IL-4, IL-7, IL-9, IL-15, and IL-21 receptors; JAK3 binds to *γ* chain and JAK1 to the other chain	Haan 2011 (A19)
RHOA signaling	Ras homolog family member A (RHOA) is a molecular switch that is activated in response to binding of chemokines, cytokines, and growth factors, and regulates activation of cytoskeletal proteins and other factors	Bros 2019 (A20)
Myelination signaling	Signaling of Wnt/*β*-catenin, PI3K/AKT/mTOR, and ERK/MAPK oligodendrocyte precursor cell differentiation and myelination/remyelination regulation	Gaesser 2016 (A21)
Mouse embryonic stem cell pluripotency	Noncoding RNAs and regulation of chromatin packing dynamics by histone modifications and DNA methylation play a vital role in pluripotency maintenance	Chen 2016 (A22)
Human embryonic stem cell pluripotency	Combination of intrinsic and extrinsic signaling pathways that regulates self-renewal of human embryonic stem cell	Mohammadi 2020 (A23)
Chronic myeloid leukemia signaling	Proliferation, self-renewal, and survival of normal and malignant stem cells	Moradi 2019 (A24)
Caudal-related homeobox transcription (CDX) gastrointestinal cancer signaling	Intestine-specific nuclear transcription factor, strongly implicated in multiple tumorigenesis	Yu 2019 (A25)
Axonal guidance signaling	Axonal guidance signaling-associated pathways (including NGF and semaphorin 3A) are suppressed in CRSwNP	Wu 2018 (A26)
Pathways associated with Factor 2		
Role of JAK2 in hormone-like cytokine signaling	JAK2 is essential for signaling through hormone-like cytokines and growth factors such as interleukin-3 (IL-3), IL-5, granulocyte macrophage-colony stimulating factor (GM-CSF), erythropoietin (EPO), and thrombopoietin	Wang 2016 (A27)
Osteoarthritis	Related to pathological signaling pathways, such as Wnt/β-catenin, NF-*κ*B, focal adhesion, hypoxia inducible factor (HIFs), TGFβ, and other pathways and the key regulators AMPK, mTOR, and RUNX2	Yao 2023 (A28)
Myelination signaling	Signaling of Wnt/β-catenin, PI3K/AKT/mTOR regulators, and ERK/MAPK	Gaesser 2016 (A29)
Human embryonic stem cell pluripotency	Intrinsic and extrinsic signaling pathways that regulate self-renewal of human embryonic stem cell	Mohammadi 2020 (A30)
Hepatic fibrosis/Hepatic stellate cell activation	IL-17 directly induces production of collagen type I in hepatic stellate cells by activating signal transducer and activator of transcription 3 (STAT3) signaling pathway	Meng 2012 (A31)
Gustation	In CRSwNP, downregulated genes are predominantly enriched for gustatory sensory perception, tissue homeostasis, and muscle system process	Wang 2022 (A32)
Cardiac hypertrophy signaling (enhanced)	Cellular metabolism, proliferation, non-coding RNAs, immune responses, translational regulation, and epigenetic modifications, regulating cardiac hypertrophy	Nakamura 2018 (A33)
CREB signaling in neurons	Intracellular protein that regulates the expression of genes that are important in dopaminergic neurons	Wang 2018 (A34)
CMP-N-acetyl-neuraminate biosynthesis I	CMP-N-acetylneuraminate synthetase (CMAS) is a key enzyme in sialic acid incorporation pathway, and is crucial in the virulence and survival of several pathogenic bacteria	Bose 2019 (A35)
Androgen biosynthesis	Hyperandrogenism can activate mononuclear cells (MNC) in the fasting state, increasing MNC sensitivity to glucose	Gonzalez 2011 (A36)
Pathways associated with Factor 3		
TREM1 signaling	Triggering receptor expressed on myeloid cells- 1 (TREM1). Neutrophil Activation Pathway is suppressed in eosinophilic nasal polyps	Wu 2018 (A37)
Superpathway of cholesterol biosynthesis; Cholesterol I, II (via 24,25-dihydrolanosterol); III (via desmosterol)	Cholesterol and cholesterol derivatives shape plasma membrane fluidity and lipid raft dynamics, affecting the formation of the immunological synapse and its downstream signalling events, modulating T-cell activation and function	Cardoso 2021 (A38)
Role of cytokines in mediating communication between immune cells	In Th2-polarized environment of allergic asthma, high IL-4 levels produced by locally infiltrating innate lymphoid cells and helper T cells promote an alternatively activated M2a phenotype in macrophages, affecting local immune response and airway structure	Ewan 2021 (A39)
Intrinsic prothrombin activation pathway; Extrinsic prothrombin activation pathway; Coagulation system	Activation of the coagulation pathway, including increased thrombin-antithrombin and D-dimer, has been demonstrated in chronic urticaria	Kim 2015 (A40)
Airway inflammation asthma	Type 2 inflammation pathways link the pathogenesis of asthma and CRSwNP	Laidlaw 2020 (A41)
Pathways associated with Factor 4		
Wound healing signaling pathway	Involvement of JAK/STAT signaling in chronic wounds	Jere 2017 (A42)
Role of osteoclasts in rheumatoid arthritis signaling pathway	IL-1β, IL-6, TNF-α, IL-17 and hypoxia-inducible factor-1α (HIF-1α) are produced that could mediate bone loss	Hu 2022 (A43)
Phagosome maturation	Regulated by p38 mitogen-activated protein kinase (MAPK), which is activated by TLRs	Blander 2004 (A44)
Mineralocorticoid biosynthesis	Mineralocorticoid receptor activation result in increased tissue oxidative stress and vascular inflammation	Young 2008 (A45)
Microautophagy signaling pathway	During lysosomal inhibition, MyD88 is accumulated, and overabundant MyD88 autoactivates downstream signaling or enhance TLR/IL-1R-mediated signaling	Into 2017 (A46)
Huntington disease signaling	RhoA regulation and downstream cellular functions, and signaling in neurodegenerative diseases	Schmidt 2022 (A47)
Hepatic fibrosis/Hepatic stellate cell activation	IL-17 directly induced production of collagen type I in hepatic stellate cells by activating the signal transducer and activator of transcription 3 (STAT3) signaling pathway	Meng 2012 (A48)
Glucocorticoid biosynthesis	Elevated IL-17A level promotes pyroptosis in hNECs through the ERK-NLRP3/caspase-1 signaling pathway and contributes to glucocorticoid resistance by affecting glucocorticoid receptor homeostasis in CRSwNP	Li 2022 (A49)
CSDE1 signaling pathway	Cold shock domain-containing E1 is an RNA-binding protein that can directly interrupt transcription and translation of proteins and has been shown to prevent neurogenesis in human embryonic stem cells	Lee 2017 (A50)
Assembly of RNA polymerase I complex	RNA polymerase I and RNAPIII are protein complexes specializing in transcription of highly abundant non-coding RNAs, such as ribosomal RNA and transfer RNA	Turowski 2021 (A51)
Pathways associated with Factor 5		
Wound healing signaling pathway	JAK/STAT signaling and PBM in chronic wounds	Jere 2017 (A52)
Role of osteoclasts in rheumatoid arthritis (RA) signaling path	IL-1β, IL-6, TNF-α, IL-17 and hypoxia-inducible factor-1α (HIF-1α) are produced that could mediate bone loss	Hu 2022 (A53)
Role of osteoblasts in RA signaling pathway	Involves proinflammatory cytokines Tumor Necrosis factor-α, Interleukin-1	Hu 2022 (A53)
Pulmonary fibrosis idiopathic signaling pathway	Primary human fibroblast cultures signaling leads to IL-6R overexpression. The IL-6/STAT3/Smad3 axis facilitates cellular responses and fibrotic disease	Shochet 2020 (A54)
Pathogen induced cytokine storm signaling pathway	Toll-like receptor-4 (TLR4) signaling activates diverse transcription factors and induces proinflammatory cytokine expression	Kobayashi 2013 (A55)
Neutrophil extracellular trap signaling pathway	Triggered by innate immune receptors through downstream intracellular signaling, which activate myeloperoxidase, neutrophil elastase, and protein-arginine deiminase type 4 to promote chromatin decondensation	Papayannopoulos 2018 (A56)
Microautophagy signaling pathway	During lysosomal inhibition, MyD88 is accumulated, and overabundant MyD88 autoactivates downstream signaling or enhance TLR/IL-1R-mediated signaling	Into 2017 (A57)
Iron homeostasis signaling pathway	IL-33 is associated with erythrocytes and heme to promote the generation of mature splenic red pulp macrophages through activation of the MyD88 adaptor protein and ERK1/2 kinases downstream of IL-33 receptor, IL1RL1	Lu 2020 (A58)
Hepatic fibrosis/Hepatic stellate cell activation	IL-17 directly induced production of collagen type I in hepatic stellate cells by activating the signal transducer and activator of transcription 3 (STAT3) signaling pathway	Meng 2012 (A59)
GP6 signaling pathway	GP6 is a collagen and fibrin receptor for tissue repair, wound healing, general inflammation, and innate immunity	Nurden 2019 (A60)

### 3.3 Correlation of factor variation with clinical features

**Figure 4:**
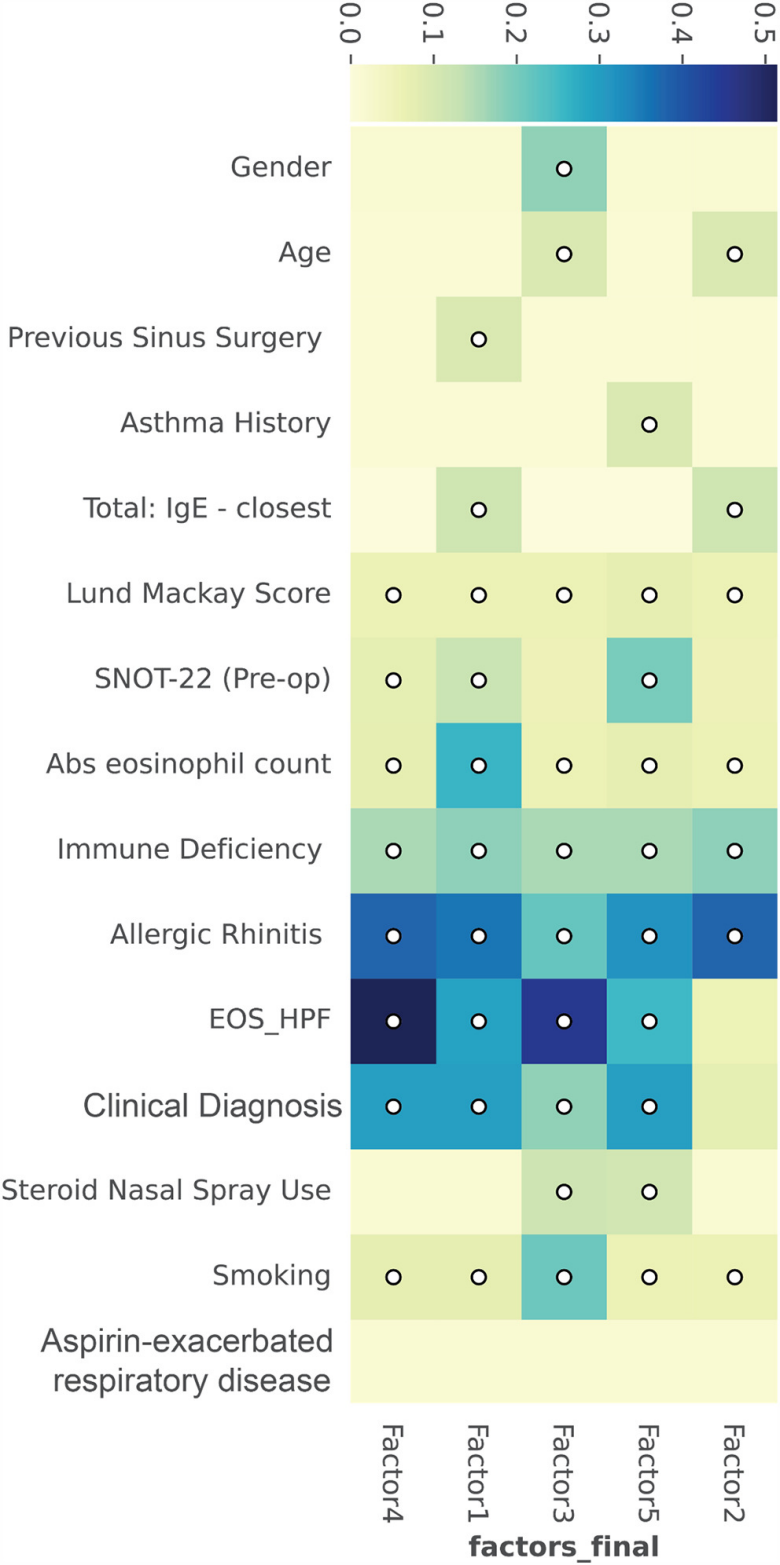
Hierarchical-all-against-all clustering used to represent the correlation between factor distribution and clinical metrics of samples. Similarly, behaving factors and clinical metrics are binned into clusters. Correlation is shown from high (blue) to low (beige).

The five factors of variation were correlated with clinical features using HAllA (Figure 4). Significant correlations for Factor 1 were with allergic rhinitis, absolute blood eosinophil count, tissue eosinophil counts, and clinical diagnosis. For Factor 2, the most significant correlations were allergic rhinitis and immune deficiency. Factor 3's correlations were tissue eosinophil counts, smoking, allergic rhinitis, and gender. Factor 4's most significant correlations were tissue eosinophil counts, allergic rhinitis, and clinical diagnosis. Factor 5's most significant correlations were allergic rhinitis, clinical diagnosis, tissue eosinophil counts, and pre-operative SNOT-22. Tissue eosinophil counts mostly correlated with Factors 4 and 3, and absolute blood eosinophil counts only significantly correlated with Factor 1. Allergic rhinitis strongly correlated with Factors 1, 2, 4, and 5, and moderately correlated with Factor 3. Age, previous sinus surgery, asthma history, total IgE, CT score, steroid nasal spray use, and AERD presented with weak correlations.

### 3.4 Examination of sample distribution across factors: tissue eosinophilia was able to better cluster subjects in two distinct groups compared to phenotypic status

**Figure 5:**
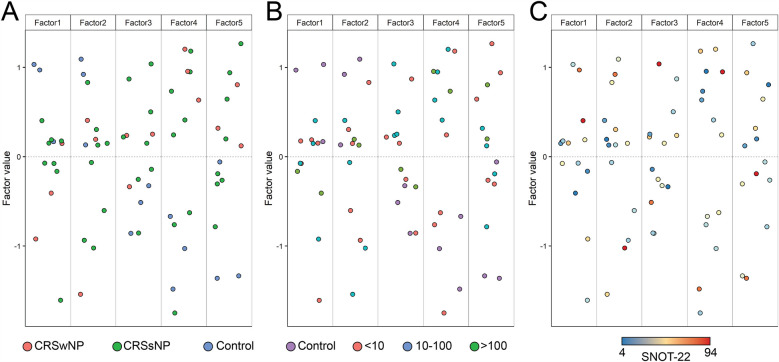
Sample distribution across all five factors colored by **(A)** diagnosis/polyp status, **(B)** tissue eosinophil numbers/hpf, and **(C)** SNOT-22 scores.

We examined sample distribution across all five factors identified with JDR. Figure 5 (A, B, C) shows the sample distribution colored by diagnosis, tissue eosinophils/hpf, and SNOT-22 scores, respectively. Whereas SNOT-22 seemed to lead to a random distribution, both clinical diagnosis (CRSwNP, CRSsNP vs. control) and tissue eosinophils were able to better cluster subjects in two distinct groups. This was especially evident for factor 4, where all 3 controls were separated from CRSwNP and/or CRS ≥10 eos/hpf.

### 3.5 DNA methylation and mRNA heatmaps failed to cluster CRSwNP and CRSsNP separately

**Figure 6:**
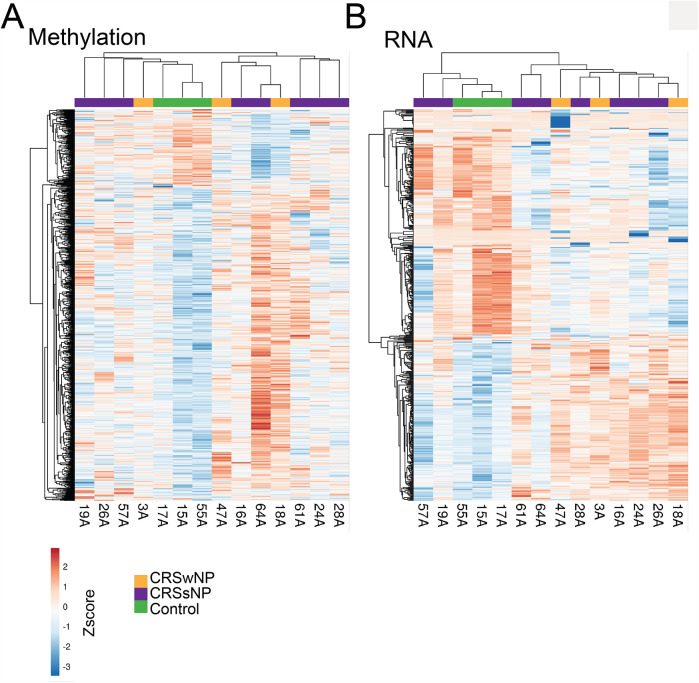
Heatmaps of the association between each subject (*x*-axis) with **(A)** DNA methylation and **(B)** RNA expression on the *y*-axis. Color key for Subjects: CRSwNP is orange, CRSsNP is purple, and Controls are green. Within the heatmap, red represents higher hypermethylation and mRNA expression.

The association of each cytokine with DNA methylation and mRNA expression in each subject was examined. DNA methylation (Figure 6A) and mRNA heatmaps (Figure 6B) showed all 3 control samples clustered together. There was no clear clustering observed for phenotypical subtypes of CRS by methylation and mRNA expression status, likely exposing the limitations of classifying only by polyp status.

### 3.6 Correlation between DNA, RNA, and cytokine expression: Two distinct clusters of cytokines were noted, with opposed positive, neutral, and negative correlations for cytokines

**Figure 7:**
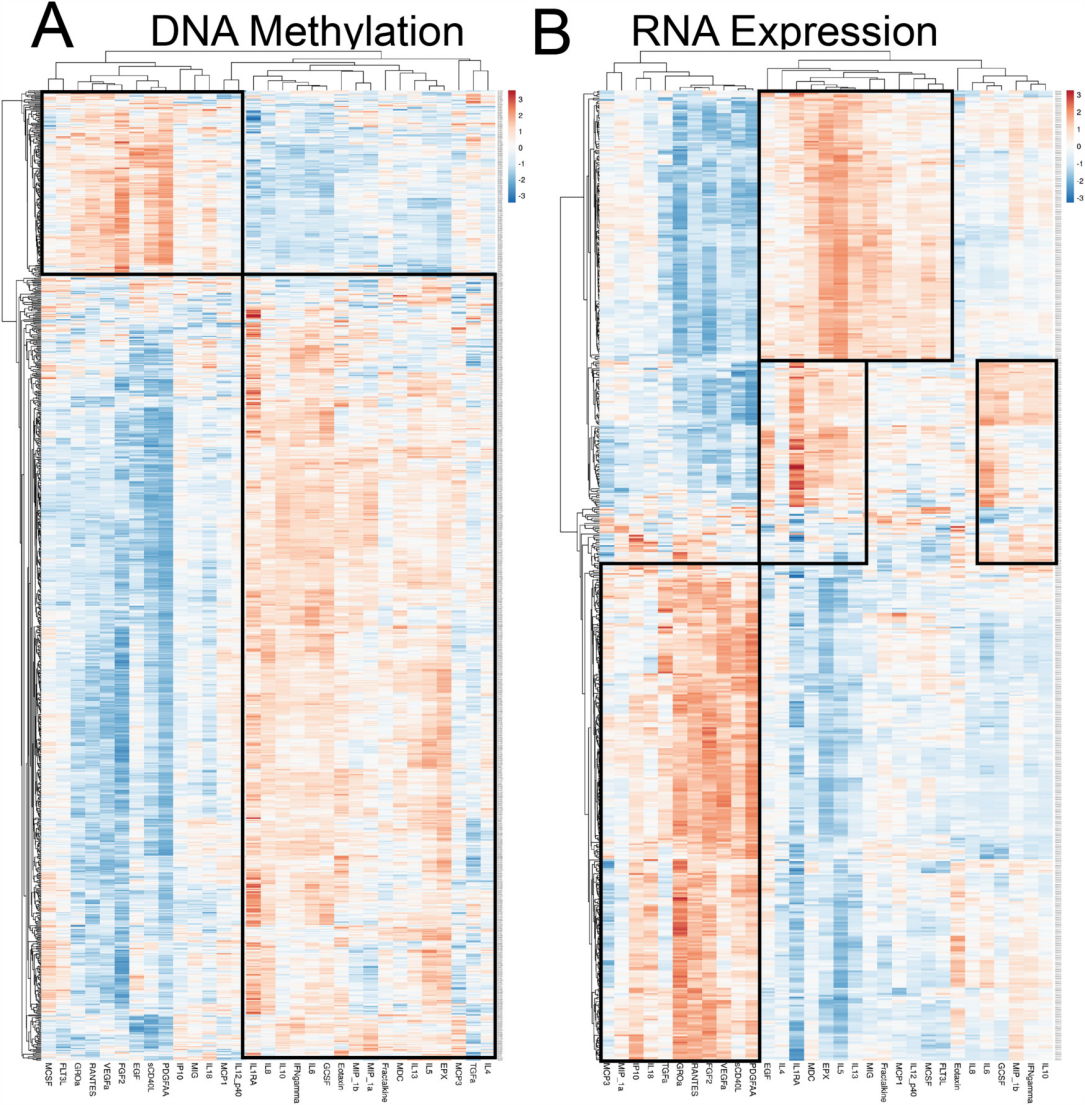
Heatmap of **(A)** differential DNA methylation and **(B)** differential RNA expression to show correlations to cytokines. The *y*-axis represents the genes associated with the DMRs/ mRNAs, and the *x*-axis represents individual cytokines. Red: strong correlation; blue: weak correlation; white: no correlation.

Next, we investigated the correlation between DNA, RNA, and cytokine expression and identified two distinct clusters with opposed positive, neutral, and negative correlations for the cytokine-methylation analysis (Figure 7A). The first cluster included MCSF, FLT3l, GROa, RANTES, VEGFa, FGF2, EGF, sCD40l, PDGFAA, IP10, MIG, IL-18, MCP1, and IL-12p40. The second cluster included IL-4, IL-13, IL-5, IL-1RA, IL-8, IL-10, INF*γ*, IL-6, G-CSF, Eotaxin, MIP-1b, MIP-1a, Fractalkine, MDC, EPX, MCP3, and TGF*α*. Two distinct clusters of cytokines were noted with opposed positive, neutral, and negative correlations for cytokines-RNA expression analysis as well (Figure 7B). The first cluster included MCP3, MIP-1a, IP10, IL-18, TGF*α*, GROa, RANTES, FGF2, VEGFa, sCD40l, and PDGFAA. The second cluster included EGF, IL-4, IL-1RA, MDC, EPX, IL-5, IL-13, MIG, Fractalkine, MCP1, IL-12p40, MCSF, FLT3l, Eotaxin, IL-8, IL-6, GCSF, MIP-1b, IFN*γ*, and IL-10.

### 3.7 Associations of individual cytokines with upstream DNA methylation and RNA expression were found

**Figure 8:**
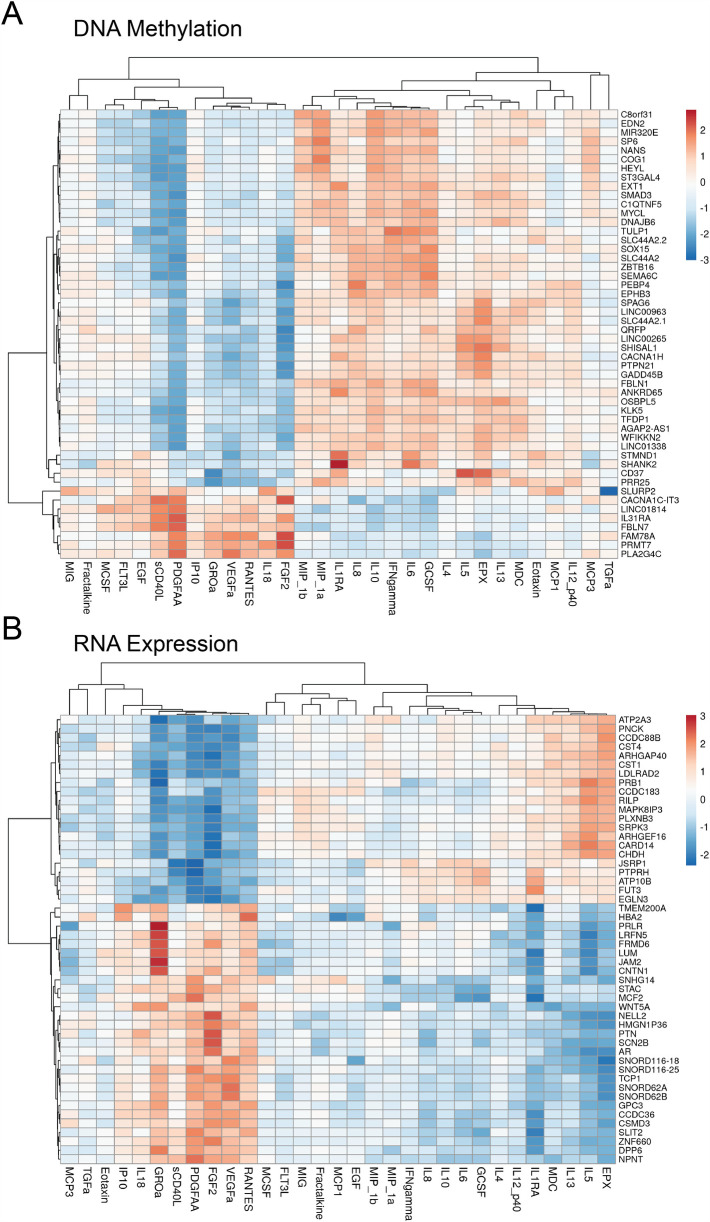
Heatmap correlating each cytokine (*x*-axis) with their top 50 genes with **(A)** differential DNA methylation & **(B)** differentially expressed RNA (*y*-axis).

Isolated cytokine analysis was used next to study associations between cytokine, DNA, and RNA. The analysis revealed that IL-5 was associated with 720 differentially expressed (DE) RNAs and 172 differentially methylated regions (DMRs) on the DNA. IL-13 associated with 49 DE-RNAs and 180 DMRs, IL-10 to 54 DE-RNAs and 82 DMRs, IFN*γ* to 71 DE-RNAs and 123 DMRs, and IL-6 to 236 DE-RNAs and 178 DMRs. IL-4 and TGF did not significantly correlate with the other data modalities. Figures 8A,B illustrate the top 50 differentially methylated genes and differentially expressed mRNAs, respectively, for each of the 30 cytokines. Table 5 presents the top 10 DMRs and the differentially expressed mRNA identified in our study as related to many of these cytokines.

**Table 5:** Top 10 differentially methylated DNA as well as differentially expressed mRNA molecules.

Cytokines	Differentially methylated DNA	Differentially expressed mRNA
IL-1RA	SHANK2, STMND1, CD37, ANKRD65, EXT1, DNAJB6, SOX15, PRR25, TULP1, C1QTNF5	FUT3, ATP10B, PTPRH, ATP2A3, CHDH, PLXNB3, PRB1, LDLRAD2, EGLN3, SRPK3
IL-4	OSBPL5, TFDP1, AGAP2-AS1, ANKRD65, SHISAL1, LINC00265 CD37, STMND1, EXT1, QRFP	CST4, ARHGAP40, ARHGEF16, CARD14, FUT3, EGLN3, PLXNB3, PTPRH, ATP10B, CST1
IL-5	CD37, CACNA1H, LINC00265 SHISAL1, PTPN21, LINC00963, GADD45B, SLC44A2.1, QRFP, OSBPL5	PRB1, CARD14, RILP, ARHGEF16, CHDH, CCDC183, MAPK8IP3, SRPK3, ARHGAP40, PNCK
IL-6	SHANK2, STMND1, TULP1, SLC44A2.2, C1QTNF5, ANKRD65, FBLN1, ZBTB16, MIR320E, EPHB3	JSRP1, PTPRH, ATP10B, FUT3, EGLN3, FRMD6, LFRN5, ATP2A3, PNCK, CCDC88B
IL-8	PEBP4, EPHB3, SOX15, TULP1, QRFP, STMND1, GADD45B, PTPN21, LINC00265, SPAG6	ATP10B, EGLN3, PTPRH, FUT13, PRLR, CHDH, JSRP1, SRPK3, ARHGAP40, PNCK
IL-10	C8ORF31, EDN2, MIR320E, LINC01338, KLK5, FBLN1, ZBTB16, COG1, SOX15, HEYL	JSRP1, PTPRH, EGLN3, ATP10B, ATP2A3, PNCK, CCDC88B, CST4, ARHGAP40, CST1
IL-12p40	PEBP4, PRR25, EPHB3, SPAG6, SLC44A2.1, QRFP, LINC00265, C8ORF31, MIR320E, CD37	EGLN3, FUT3, CHDH, MAPK8IP3, CCSC183, LDLRAD2, CST1, ARHGEF16, PNCK, CCDC88B
IL-13	OSBPL5, SHISAL1, DNAJB6, CD37, SMAD3, ST3GAL4, WFIKKN2, TFDP1, QRFP, AGAP2-AS1	CARD14, ARHGEF16, RILP, PRB1, ARHGAP40, ATP2A3, CST4, CST1, SRPK3, CHDH
EPX	CD37, SHISAL1, SLC44A2.1, SPAG6, LINC00963, CACNA1H, PTPN21, GADD45B, WFIKKN2, AGAP2-AS1	CCDC88B, PNCK, PRB1, SRPK3, CHDH, CARD14, CST4, RILP, CCDC183, CST1
INF-γ	TULP1, SLC44A2.2, SOX15, SLC44A2, ZBTB16, SEMA6C, PEBP4, MIR320E, EPHB3, NANS	JSRP1, PTPRH, ATP10B, FUT3, EGLN3, LRFN5, JAM2, PRLR, HBA2, FRMD6
TGF-alpha	FAM78A, IL31RA, ANKRD65, EXT1, SOX15, SP6, DNAJB6, CACNA1C-IT3, SHANK2, SLC44A2	HBA2, NELL2, HMGN1P36, SNORD116-18, TCP1, SNORD116-25, ATP10B, PTPRH, FUT3, SNORD62A

### 3.8 Conjoint cytokine analyses identified common upstream DNA methylation and RNA expression for some cytokines

**Figure 9:**
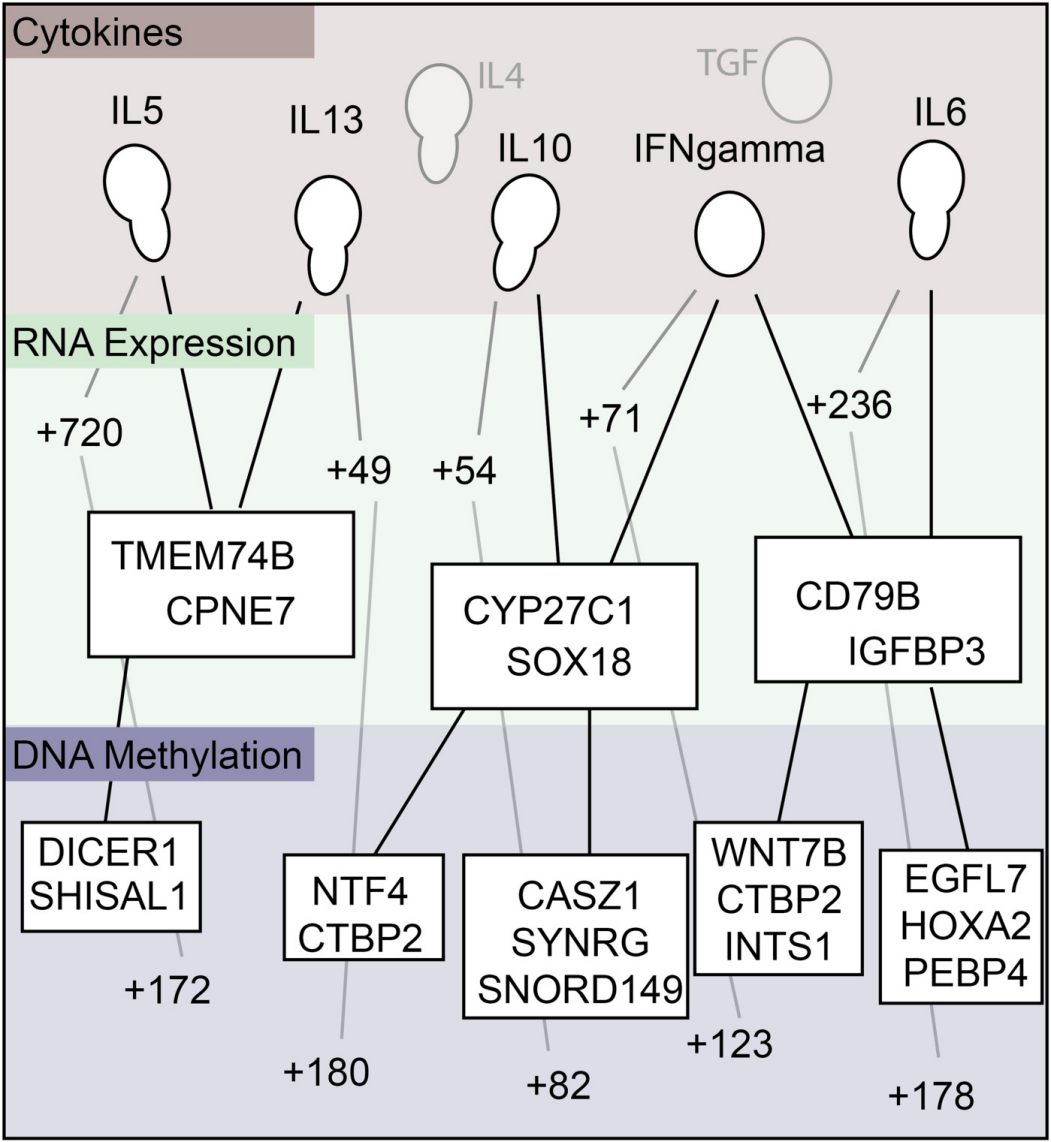
Summary map relating cytokine to RNA expression, and RNA expression to DNA methylation.

Next, conjoint cytokine analysis was performed (Figure 9) to identify commonly shared genes. The conjoint analysis showed that cytokines IL-5 and IL-13 were similarly correlated with RNA expression of TMEM74B and CPNE7, and with DNA methylation of DICER1 and SHISAL1. IL-10 and IFN*γ* were correlated to RNA expression of CYP27C1 and SOX18, and with DNA methylation of CASZ1, SYNRG, SNORD149, NTF4, and CTBP2. IFN*γ* and IL-6 were similarly correlated to RNA expression of CD79B and GFBP3, and with DNA methylation of EGFL7, HOXA2, PEBP4, WNT7B, CTBP2, and INTS1 (Figure 9). Table 6 enlists the function of genes identified on conjoint cytokine analysis.

**Table 6:** Function of genes identified on conjoint cytokine analysis.

Gene	Name	Function	Cytokine
Differentially Methylated Genes			
DICER1	Dicer 1, ribonuclease III	The encoded protein functions as a ribonuclease, and is a strong antiviral agent active against RNA viruses, including Zika and SARS-CoV-2 viruses	IL-5 and IL-13
SHISAL1	Shisa like 1	Predicted to be integral component of membrane	IL-5 and IL-13
NTF4	Neurotrophin factor 4	Neurotrophins control survival and differentiation of mammalian neurons	IL-10 and IFNγ
CTBP2	C-terminal binding protein 2	This gene can encode two distinct proteins: one isoform is a transcriptional repressor, while the other is a component of specialized synapses known as synaptic ribbons. Both proteins contain a NAD + binding domain similar to NAD + -dependent 2-hydroxyacid dehydrogenases.	IL-10 and IFNγ as well as IFNγ and IL-6
CASZ1	Castor zinc finger 1	The protein encoded by this gene is a zinc finger transcription factor and may function as a tumor suppressor	IL-10 and IFNγ
SYNRG	Synergin gamma	Encodes a protein that interacts with the gamma subunit of AP1 clathrin-adaptor complex	IL-10 and IFNγ
SNORD 149	Small Nucleolar RNA, 149	*snoRNA (non-protein-coding); Small nucleolar RNAs (snoRNAs) are a class of small RNA that assist in chemical modifications of other RNAs, such as ribosomal RNAs	IL-10 and IFNγ
WNT7B	Wnt family member 7b	Member of the WNT gene family, which consists of structurally related genes encoding secreted signaling proteins regulating cell fate and patterning	IFNγ and IL-6
INTS1	Integrator complex subunit 1	Is a subunit of the Integrator complex, which associates with the RNA polymerase II large subunit and mediates processing of small nuclear RNAs U1 and U2.	IFNγ and IL-6
EGFL7	Epidermal Growth Factor like domain multiple 7	Encodes a secreted endothelial cell protein that contains two epidermal growth factor-like domains. The encoded protein may play a role in regulating vasculogenesis, and growth and proliferation of tumor cells.	IFNγ and IL-6
HOXA2	Homeobox A2	Encodes a DNA-binding transcription factor regulating gene expression, morphogenesis, and differentiation	IFNγ and IL-6
PEBP4	Phosphatidylethanolamine binding protein 4	Phosphatidylethanolamine (PE)-binding proteins, including PEBP4, are an evolutionarily conserved family of proteins with pivotal biologic functions, such as lipid binding and inhibition of serine proteases	IFNγ and IL-6
Differentially Expressed mRNA			
TMEM74B	Transmembrane protein 74B	Predicted to be integral component of membrane.	IL-5 and IL-13
CPNE7	Castor zinc finger 1	Encodes a zinc finger transcription factor. The encoded protein may function as a tumor suppressor gene.	IL-5 and IL-13
CYP27C1	Cytochrome P450 family 27 subfamily C member 1	Encodes a member of the cytochrome P450 superfamily of enzymes which are monooxygenases catalyzing many reactions involved in drug metabolism and synthesis of cholesterol, steroids, and other lipids.	IL-5 and IL-13
SOX18	SRY-box transcription factor 18	This gene encodes a member of the SOX (SRY-related HMG-box) family of transcription factors. The encoded protein may function as a transcriptional regulator and play a role in blood vessel and lymphatic vessel development.	IL-10 and IFNγ
CD79B	CD79b molecule	Gene encodes Ig-beta protein of B-cell antigen component. It associates with Ig-alpha and Ig-beta, necessary for expression and function of B-cell antigen receptor	IFNγ and IL-6
IGFBP3		Insulin-like growth factor binding protein 3; this gene is a member of the IGFBP family. It prolongs half-life of IGFs and alters their interaction with cell surface receptors.	IFNγ and IL-6

Sources: National Institutes of Health National Library of Medicine, National Center for Biotechnology Information https://www.ncbi.nlm.nih.gov/gene National Human Genome Research Institute https://www.genome.gov/genetics-glossary/Pseudogene Last accessed August 16, 2024.

*snoRNA: small nucleolar RNA.

## 4 Discussion

The results of our study support our hypothesis that environmental insults may be significant in CRS pathogenesis through epigenetic mechanisms that result in dysregulated mRNA transcription and cytokine production downstream. Chronic dysregulated immune responses may continue long past the initial external insult through the induction of epigenetic changes, as seen in CRS (5).

Although the changes that occur at the histopathological and cytokine/protein levels have recently become better characterized in subjects with CRS (35–37), the genetic mechanism associated with such changes has not been fully characterized (3). In a sparse area of research, this study provides the first multi-omics analysis of CRS tissue from the United States, validating the association of epigenetic changes with transcriptomic and proteomic signatures seen in CRS. Furthermore, multi-omics analysis using DNA methylation, mRNA expression, and cytokine expression datasets successfully separated clusters of control and CRS subjects, demonstrating the utility of multi-omics analysis as a valuable tool in studying CRS. Our study is novel in using a multi-omics integration of DNA, RNA, and cytokine data to study CRS. Only two prior multi-omics studies have investigated CRS, but neither studied DNA data, and both were conducted outside of North America (18, 38). Miyata et al. (38), isolated eosinophils from six nasal polyp patients and performed multi-omics analysis using lipidomics, proteomics, and transcriptomics. Hoggard et al. (18), investigated temporal changes in polyp tissue in CRS in response to systemic corticosteroids in three males with CRSwNP subjects who underwent surgery, assessing natural variability over time and local response to systemic corticosteroid therapy. The authors found that the most highly abundant transcripts and proteins were associated with pathways involved in inflammation, FAS, cadherin, integrin, Wnt, apoptosis, cytoskeletal signaling, coagulation, and B- and T-cell activation. Given that DNA methylation was the most significant data modality contributing to the total variance between CRS and control subjects, epigenetic modifications are critical for further study in CRS for mechanistic and therapeutic targets. In addition, epigenetic mechanisms help explain shifts in the dominant CRS inflammatory pattern from non-type 2 to type 2, as is being noted in Asian regions as they undergo industrialization (36, 37, 39).

Our study further identified several known and potential mechanistic pathways and proteins involved in immunity and structural integrity, which may have roles in CRS pathogenesis. These are related to cytokine signal transduction, granule fusion events, phagosome maturation, toll-like receptors (TLRs) activation, reactive oxygen species formation, cellular metabolism, translational regulation, etc. The identification of JAK signaling also highlights the potential therapeutic role of JAK inhibitors in recalcitrant CRS, like current trials for asthma therapy (40). Many novel DMRs and DE mRNA (Tables 4A, B) were identified, including genes involved in membrane stability, homeostasis, as well as the gustation pathway, which are targets for further research (Supplementary Table S1).

Novel findings on conjoint cytokine analysis (Figure 9) showed that the cytokines IL-5 and IL-13 shared genes with RNA expression of TMEM74B and CPNE7, and with DNA methylation of DICER1 and SHISAL1. We also similarly noted shared genes for IL-10 and IFN*γ*, as well as IFN*γ* and IL-6. Table 6 details the functions of these genes. Both IL-5 and IL-13 are well recognized for their roles in the type-2 inflammatory process predominantly associated with CRSwNP, and hence, their association in differentially regulated upstream DNAs and RNAs is understandable, further reinforcing the utility of the multiomics approach. IFN*γ* is detected at lower levels in CRS tissue, reducing the antiviral immune response, which could result in or exacerbate the CRS following a viral infection (41). The role of IL-10 and IL-6 is reported in the literature (42, 43), and their association with IFN*γ* is interesting. DMRs and DE RNAs identified in association with key inflammatory cytokines involved in CRS pathogenesis, like IL-5, IL-13, IL-10, IFN*γ*, and IL-6 (Table 5), could be potentially important future therapeutic targets.

### 4.1 Limitations

This is a small study, albeit with the largest number of subjects published for CRS multiomics. While the multiomics approach distinguished two clusters, one of which was composed entirely of CRS patients, the other grouped three controls and two non-eosinophilic CRSsNP subjects, and perhaps these may have been clustered differently in a larger sample size. Genomic assays and multiomics analysis are prohibitively expensive, complex, and require technical expertise in integration, statistics, and systems biology. However, we hope that with reduced cost of genomic assays and multiomics analysis and support from extramural funding, larger prospective sampling can be performed for future studies.

Prospective, longitudinal studies with sample collection at multiple time points are needed to study CRS disease evolution. RNA expression is transient and may not correlate with protein level unless analyzed concurrently, which was mitigated by collecting tissue for histopathology, DNA methylation, and cytokine assay simultaneously in this study.

The multi-omics approach may also allow for focused upstream gene profiling of targeted cytokines of interest, such as IL-4, IL-5, IL-13, and others, in addition to an unsupervised approach that was used in this study. We anticipate that the use of single-cell RNA sequencing may be necessary for this approach rather than the bulk tissue sample that was used for this study.

Technical limitations in the study include bulk tissue RNA sequencing, which can only provide an average gene expression profile for the entire sample, but is cheaper than single-cell RNA sequencing (scRNA-seq) while identifying global differences in gene expression between disease and control states. Additionally, of the multiplex assay performed for cytokines, only 30 could be included per study methodology for multiomics analysis. Quantifying tissue eosinophilia with histopathology is imperfect, as degranulated eosinophils are difficult to measure. More sensitive novel assays, such as those from NanoString Technology (https://nanostring.com/), are planned for future study.

## 5 Conclusions

The study supports the hypothesis that environmental insults may be significant drivers of CRS pathogenesis through epigenetic mechanisms that result in dysregulated mRNA transcription and cytokine expression. The most novel part of this study is the integration of epigenetic (DNA methylation), transcriptomic (mRNA), and proteomic (cytokine) data to uncover novel insights into the pathogenesis of CRS. This multi-omics approach is the first of its kind to study environment-host interactions in CRS etiopathogenesis. The multi-omics analysis clearly separated clusters of control and CRS subjects, demonstrating its validity in future research. DNA methylation also contributed most to total variance, underscoring the role of environmental factors in CRS. Key cytokines like IL-5, IL-13, IL-10, IFN*γ*, and IL-6 were associated with hundreds of differentially methylated regions (DMRs) and differentially expressed mRNAs, providing future targets for study. IL-5 and IL-13, IL-10 and IFN*γ*, and IFN*γ* and IL-6 were associated with common upstream genes. The study further identified interactions of methylated DNA, mRNA, and cytokines in CRS pathogenesis, highlighting novel molecules and pathways that may be potential therapeutic targets.

## Data Availability

The original contributions presented in the study are included in the article/supplementary material, further inquiries can be directed to the corresponding author/s.
