# Supplementary material for: Epigenetic modifications are associated with mRNA and cytokine expression changes in chronic rhinosinusitis: a multiomics study from the United States
Source: Front Allergy. 2025 Jun 5;6:1606255. doi: 10.3389/falgy.2025.1606255 (PMC12176764; doi:10.3389/falgy.2025.1606255)
Supplement: Supplementary file 1 [file Datasheet1.docx]

**SUPPLEMENT**

- 1. **METHODOLOGY**
  2. **RNA Sequencing:**
     1. *RNA isolation*: Bio-banked tissue samples were thawed for analysis. RNA concentration and quality were measured using Qubit fluorometry (Invitrogen, Carlsbad, CA) and Agilent Fragment Analyzer (Santa Clara, CA). Samples with DV200 >20% underwent library prep using Illumina TruSeq® RNA Exome Library Prep kit (San Diego, CA) with up to 1000 ng of RNA. cDNA library quality was assessed using TapeStation D1000 (Agilent, Santa Clara, CA). Transcriptome coding regions were captured by pooling up to four cDNA libraries, then analyzed with Agilent Bioanalyzer DNA 1000 chip (Santa Clara, CA) and Qubit (Invitrogen, Carlsbad, CA). Libraries were sequenced in 2 pools per lane on an Illumina HiSeq 4000 (100×2 paired-end reads) and base-calling using Illumina’s RTA v2.7.7.
     2. *Bioinformatics:* Paired-end RNA sequencing reads were processed through the RNA-Seq bioinformatics pipeline, MAP-RSeq v3.1.4^27^ which utilizes STAR^43^ alignment (hg38). Gene and exon expression quantification were performed using the Subread^44^ package to obtain raw and normalized Fragments Per Kilobase per Million (FPKM) mapped reads.
     3. *Differential expression and pathway analyses:* Differentially expressed genes (DEG) were identified from raw gene counts using edgeR 2.6.2.^28^ DEGs were reported with log2 fold change and False Discovery Rate (FDR < 5%). Canonical pathway analysis using Ingenuity pathway analysis (IPA) software (Ingenuity® Systems) identified significant pathways (p-value < 5%).
  3. **DNA Methylation:**
     1. *DNA extraction:* DNA was extracted using the Qiagen QIAamp DNA Mini kit (Reference no. 51306), a silica-membrane method. Tissues were lysed using Proteinase K and lysis buffer, bound to the membrane, washed, and eluted in 100uL of buffer AE (10 mM Tris·Cl; 0.5 mM EDTA; pH 9.0).
     2. *Reduced Representation Bisulfite Sequencing (RRBS) Library prep and Sequencing on Illumina’s HiSeq4000:* RRBS libraries were prepared using 100ng genomic DNA and the NuGen RRBS Ovation Kit (NuGen, Redwood City, CA). dsDNA was digested with Msp1, ligated with indexed methylated adaptors, repaired, and bisulfite modified using the EZ-DNA Methylation Kit (Zymo Research, Irvine, CA). The product was PCR amplified, purified with Ampure beads (Beckman Coulter, Indianapolis, IN), and analyzed using Agilent Bioanalyzer (Agilent, Santa Clara, CA) and Qubit fluorometry (Invitrogen, Carlsbad, CA). Libraries were pooled with 10% PhiX (Illumina, San Diego, CA) added to increase sequencing quality, and sequenced at ~75million reads/sample (51×2 paired-end) on an Illumina HiSeq 4000 (Illumina, San Diego, CA). Base-calling was performed using Illumina’s RTA v2.7.3.
     3. *RRBS data preprocessing & quality assessment:* RRBS data were analyzed using a streamlined analysis and annotation pipeline for reduced representational bisulfite sequencing, SAAP-RRBS.^45^ FASTQ files were trimmed to remove adaptor sequences discarding reads <15bp. Trimmed reads were aligned to hg38 using BSMAP. Samtools and custom PERL scripts were used to determine methylation and bisulfite conversion ratios. CpGs with <10× coverage, >99.9^th^ percentile coverage, or absent in >50% of samples were excluded.
     4. *Bioinformatic Analysis:* Differential methylation analysis of CpG loci was performed using the Dispersion Shrinkage for Sequencing (DSS) algorithm between time points.^46^ Mean methylation and dispersion per CpG loci were calculated, and a Wald test identified significant CpGs (p-value <5%, delta methylation >5%). Differentially methylated regions (DMRs) were identified using the callDMR function of the DSS algorithm, merging statistically significant CpGs with at least 4 CpG loci per region using the minCG parameter.
  4. **SUPPLEMENTARY TABLES**

**Supplementary Table 1A: Function of Top 50 differentially methylated DNA between Cluster 1 and Cluster 2 subjects.**

| Gene Name | Gene Description | Gene type |
| --- | --- | --- |
| C8orf31 | Chromosome 8 open reading frame 31 | Non-coding |
| EDN2 | Endothelin 2 | The gene codes for a member of endothelin protein family of secretory vasoconstrictive peptides. The preproprotein is processed to a short mature form which functions as a ligand for endothelin receptors that initiate intracellular signaling events. This gene product participates in a wide range of biological processes, such as hypertension and ovulation. Altered expression of this gene is implicated in tumorigenesis. Alternative splicing results in multiple transcript variants. |
| MIR320E | MicroRNA 320e | Non-coding RNA |
| SP6 | Sp6 transcription factor | Belongs to a family of transcription factors that contain 3 classical zinc finger DNA-binding domains consisting of a zinc atom tetrahedrally coordinated by 2 cysteines and 2 histidines (C2H2 motif). These transcription factors bind to GC-rich sequences and related GT and CACCC boxes. |
| NANS | N-acetylneuraminate synthase | This gene encodes an enzyme that functions in the biosynthetic pathways of sialic acids. In vitro, the encoded protein uses N-acetylmannosamine 6-phosphate and mannose 6-phosphate as substrates to generate phosphorylated forms of N-acetylneuraminic acid (Neu5Ac) and 2-keto-3-deoxy-D-glycero-D-galacto-nononic acid (KDN), respectively; however, it exhibits much higher activity toward the Neu5Ac phosphate product. In insect cells, expression of this gene results in Neu5Ac and KDN production. This gene is related to the E. coli sialic acid synthase gene neuB, and it can partially restore sialic acid synthase activity in an E. coli neuB-negative mutant. |
| COG1 | Component of oligomeric Golgi complex 1 | The protein encoded by this gene is one of eight proteins (Cog1-8) which form a Golgi-localized complex (COG) required for normal Golgi morphology and function. It is thought that this protein is required for steps in the normal medial and trans Golgi-associated processing of glycoconjugates and plays a role in the organization of the Golgi-localized complex. |
| HEYL | HES related family bHLH transcription factor with YRPW motif like | This gene encodes a member of the hairy and enhancer of split-related (HESR) family of basic helix-loop-helix (bHLH)-type transcription factors. The sequence of the encoded protein contains a conserved bHLH and orange domain, but its YRPW motif has diverged from other HESR family members. It is thought to be an effector of Notch signaling and a regulator of cell fate decisions. |
| ST3GAL4 | ST3 beta-galactoside alpha-2,3-sialyltransferase 4 | This gene encodes a member of the glycosyltransferase 29 family, a group of enzymes involved in protein glycosylation. The encoded protein is targeted to Golgi membranes but may be proteolytically processed and secreted. The gene product may also be involved in the increased expression of sialyl Lewis X antigen seen in inflammatory responses. |
| EXT1 | Exostosin glycosyltransferase 1 | This gene encodes an endoplasmic reticulum-resident type II transmembrane glycosyltransferase involved in the chain elongation step of heparan sulfate biosynthesis. Mutations in this gene cause the type I form of multiple exostoses. |
| SMAD3 | SMAD family member 3 | The SMAD family of proteins are a group of intracellular signal transducer proteins similar to the gene products of the Drosophila gene 'mothers against decapentaplegic' (Mad) and the C. elegans gene Sma. The SMAD3 protein functions in the transforming growth factor-beta signaling pathway and transmits signals from cell surface to the nucleus, regulating gene activity and cell proliferation. This protein forms a complex with other SMAD proteins and binds DNA, functioning both as a transcription factor and tumor suppressor. Mutations in this gene are associated with aneurysms-osteoarthritis syndrome and Loeys-Dietz Syndrome 3. |
| C1QTNF5 | C1q and TNF related 5 | This gene encodes a member of a family of proteins that function as components of basement membranes and may play a role in cell adhesion. Mutations in this gene have been associated with late-onset retinal degeneration. The protein may be encoded by either a bicistronic transcript including sequence from the upstream membrane frizzled-related protein gene (MFRP), or by a monocistronic transcript expressed from an internal promoter. |
| MYCL | MYCL proto-oncogene, bHLH transcription factor | This gene is predicted to enable DNA-binding transcription factor activity, RNA polymerase II-specific and RNA polymerase II cis-regulatory region sequence-specific DNA binding activity. It is also predicted to be involved in regulation of transcription by RNA polymerase II. |
| DNAJB6 | DNA heat shock protein family (Hsp40) member B6 | This gene encodes a member of the DNAJ protein family. DNAJ family members are characterized by a highly conserved amino acid stretch called the 'J-domain' and function as one of the two major classes of molecular chaperones involved in a wide range of cellular events, such as protein folding and oligomeric protein complex assembly. This gene may also play a role in polyglutamine aggregation in specific neurons. |
| TULP1 | TUB like protein 1 | This gene encodes a member of the tubby-like gene family (TULPs). TULP proteins share a conserved C-terminal region of approximately 200 amino acid residues. The protein encoded by this gene is thought to play a role in the physiology of photoreceptors. Mutations in this gene are associated with recessive juvenile retinitis pigmentosa and Leber congenital amaurosis-15. |
| SLC44A2.2 | Solute carrier family 44, member 2 | This gene is predicted to enable choline transmembrane transporter activity, choline transport and transmembrane transport. It is predicted to be active in membrane. |
| SOX15 | SRY-box transcription factor 15 | This gene encodes a member of the SOX (SRY-related HMG-box) family of transcription factors involved in the regulation of embryonic development and in the determination of the cell fate. The encoded protein may function as a transcriptional regulator after forming a protein complex with other proteins. |
| SLC44A2 | Solute carrier family 44, member 2 | This gene enables choline transmembrane transporter activity and participates in choline transport and transmembrane transport. It is located in mitochondrion and plasma membrane. |
| ZBTB16 | Zinc finger and BTB domain containing 16 | This gene is a member of the Krueppel C2H2-type zinc-finger protein family. The protein is located in the nucleus, participates in cell cycle progression, and interacts with a histone deacetylase. Specific instances of aberrant gene rearrangement at this locus have been associated with acute promyelocytic leukemia. |
| SEMA6C | semaphorin 6C | This gene encodes a member of the semaphorin family. Semaphorins represent important molecular signals controlling multiple aspects of the cellular response that follows CNS injury, and thus may play a key role in neural regeneration. |
| PEBP4 | Phosphatidylethanolamine binding protein 4 | The phosphatidylethanolamine (PE)-binding proteins, including PEBP4, are an evolutionarily conserved family of proteins with pivotal biologic functions, such as lipid binding and inhibition of serine proteases. |
| EPHB3 | EPH receptor B3 | This gene encodes a receptor for ephrin-B family members. Ephrin receptors and their ligands, the ephrins, mediate numerous developmental processes, particularly in the nervous system. Ephrin-B (EFNB) class are transmembrane proteins. Ephrin receptors make up the largest subgroup of the receptor tyrosine kinase (RTK) family. |
| SPAG6 | Sperm associated antigen 6 | The protein expressed by this gene is recognized by anti-sperm antibodies from an infertile man. This protein localizes to the tail of permeabilized human sperm. Studies in mice suggest that this protein participates in sperm flagellar motility and maintenance of the structural integrity of mature sperm. |
| LINC00963 | Long intergenic non-protein coding RNA 963 | Non-coding RNA |
| SLC44A2.1 | Solute carrier family 44, member 2 | Enables choline transmembrane transporter activity. It is located in mitochondrion and plasma membrane. |
| QRFP | Pyroglutamylated RFamide peptide | This gene encodes a preproprotein that is proteolytically processed to generate multiple protein products. The encoded products are members of the RFamide family of neuropeptides, characterized by their common protein C-terminus consisting of an arginine (R) and an amidated phenylalanine (F). These products include the neuropeptides 26RFa and the N-terminally extended form, 43RFa. Both of these neuropeptides bind to the pyroglutamylated RFamide peptide receptor (QRFPR) and may regulate blood pressure, reproduction and food intake in rodents. |
| LINC00265 | Long intergenic non-protein coding RNA 265 | Non-coding RNA |
| SHISAL1 | Shisa like 1 | Predicted to be integral component of membrane. |
| CACNA1H | Calcium voltage-gated channel subunit alpha1 H | This gene encodes a T-type member of the alpha-1 subunit family, a protein in the voltage-dependent calcium channel complex. Calcium channels mediate the influx of calcium ions into the cell upon membrane polarization and consist of a complex of alpha-1, alpha-2/delta, beta, and gamma subunits in a 1:1:1:1 ratio. The alpha-1 subunit has 24 transmembrane segments and forms the pore through which ions pass into the cell. There are multiple isoforms of each of the proteins in the complex, either encoded by different genes or the result of alternative splicing of transcripts. Alternate transcriptional splice variants, encoding different isoforms, have been characterized for the gene. |
| PTPN21 | Protein tyrosine phosphatase non-receptor type 21 | The protein encoded by this gene is a member of the protein tyrosine phosphatase (PTP) family. PTPs are known to be signaling molecules that regulate a variety of cellular processes including cell growth, differentiation, mitotic cycle, and oncogenic transformation. This PTP contains an N-terminal domain, similar to cytoskeletal- associated proteins including band 4.1, ezrin, merlin, and radixin. This PTP was shown to specially interact with BMX/ETK, a member of Tec tyrosine kinase family characterized by a multimodular structures including PH, SH3, and SH2 domains. The interaction of this PTP with BMX kinase was found to increase the activation of STAT3, but not STAT2 kinase. Studies of the similar gene in mice suggested the possible roles of this PTP in liver regeneration and spermatogenesis. |
| GADD45B | Growth arrest and DNA damage inducible beta | This gene is a member of a group of genes whose transcript levels are increased following stressful growth arrest conditions and treatment with DNA-damaging agents. The genes in this group respond to environmental stresses by mediating activation of the p38/JNK pathway. This activation is mediated via their proteins binding and activating MTK1/MEKK4 kinase, which is an upstream activator of both p38 and JNK MAPKs. The function of these genes or their protein products participates in the regulation of growth and apoptosis. These genes are regulated by different mechanisms, but they are often coordinately expressed and can function cooperatively in inhibiting cell growth. |
| FBLN1 | Fibulin 1 | Fibulin 1 is a secreted glycoprotein that becomes incorporated into a fibrillar extracellular matrix. Calcium-binding is apparently required to mediate its binding to laminin and nidogen. It mediates platelet adhesion via binding fibrinogen. |
| ANKRD65 | Ankyrin repeat domain 65 | Broad expression in lung (RPKM 2.8), urinary bladder (RPKM 2.8) and 23 other tissues |
| OSBPL5 | Oxysterol binding protein like 5 | This gene encodes a member of the oxysterol-binding protein (OSBP) family, a group of intracellular lipid receptors that play a key role in the maintenance of cholesterol balance in the body. Most members contain an N-terminal pleckstrin homology domain and a highly conserved C-terminal OSBP-like sterol-binding domain. This gene has been shown to be imprinted, with preferential expression from the maternal allele only in placenta. |
| KLK5 | Kallikrein related peptidase 5 | Kallikreins are a subgroup of serine proteases having diverse physiological functions. Growing evidence suggests that many kallikreins are implicated in carcinogenesis and some have potential as novel cancer and other disease biomarkers. This gene is one of the fifteen kallikrein subfamily members located in a cluster on chromosome 19. Its expression is up-regulated by estrogens and progestins. The encoded protein is secreted and may be involved in desquamation in the epidermis. |
| TFDP1 | Transcription factor Dp-1 | This gene encodes a member of a family of transcription factors that heterodimerize with E2F proteins to enhance their DNA-binding activity and promote transcription from E2F target genes. The encoded protein functions as part of this complex to control the transcriptional activity of numerous genes involved in cell cycle progression from G1 to S phase. Alternative splicing results in multiple transcript variants. Pseudogenes of this gene are found on chromosomes 1, 15, and X. |
| AGAP2-AS1 | AGAP2 antisense RNA 1 | Non-coding RNA |
| WFIKKN2 | WAP, follistatin/kazal, immunoglobulin, kunitz, netrin domain containing 2 | The WFIKKN1 protein contains a WAP domain, follistatin domain, immunoglobulin domain, two tandem Kunitz domains, and an NTR domain. This gene encodes a WFIKKN1-related protein which has the same domain organization as the WFIKKN1 protein. The WAP-type, follistatin type, Kunitz-type, and NTR-type protease inhibitory domains may control the action of multiple types of proteases. |
| LINC01338 | Long intergenic non-protein coding RNA 1338 | Non-coding RNA |
| STMND1 | Stathmin domain containing 1 | Predicted to enable tubulin binding activity. Predicted to be involved in microtubule depolymerization; neuron projection development; and regulation of microtubule polymerization or depolymerization. Predicted to be active in cytoplasm and neuron projection. |
| SHANK2 | SH3 and multiple ankyrin repeat domains 2 | This gene encodes a protein that is a member of the Shank family of synaptic proteins that may function as molecular scaffolds in the postsynaptic density of excitatory synapses. Shank proteins contain multiple domains for protein-protein interaction, including ankyrin repeats, and an SH3 domain. This particular family member contains a PDZ domain, a consensus sequence for cortactin SH3 domain-binding peptides and a sterile alpha motif. The alternative splicing demonstrated in Shank genes has been suggested as a mechanism for regulating the molecular structure of Shank and the spectrum of Shank-interacting proteins in the postsynaptic densities of the adult and developing brain. |
| CD37 | CD37 molecule | The protein encoded by this gene is a member of the transmembrane 4 superfamily, also known as the tetraspanin family. Most of these members are cell-surface proteins that are characterized by the presence of four hydrophobic domains. The proteins mediate signal transduction events that play a role in the regulation of cell development, activation, growth and motility. This encoded protein is a cell surface glycoprotein that is known to complex with integrins and other transmembrane 4 superfamily proteins. It may play a role in T-cell-B-cell interactions. |
| PRR25 | Proline rich 25 | Unknown |
| SLURP2 | Secreted LY6/PLAUR domain containing 2 | This gene encodes a novel, secreted member of the Ly6/uPAR (LU) superfamily of proteins containing the unique three-finger LU domain. This gene is mainly expressed in epithelial cells, including skin and keratinocytes, and is up-regulated in psoriatic skin lesions, suggesting its involvement in the pathophysiology of psoriasis. Alternatively spliced transcript variants have been found for this gene. Read-through transcription from the neighboring upstream gene (LYNX1) generates naturally-occurring transcripts (LYNX1-SLURP2) that encode a fusion protein comprised of sequence sharing identity with each individual gene product. |
| CACNA1C-IT3 | CACNA1C intronic transcript 3 | Non-coding RNA |
| LINC01814 | Long intergenic non-protein coding RNA 1814 | Non-coding RNA |
| IL31RA | Interleukin 31 receptor A | The protein encoded by this gene belongs to the type I cytokine receptor family. This receptor, with homology to gp130, is expressed on monocytes, and participates in IL-31 signaling via activation of STAT-3 and STAT-5. It functions either as a monomer, or as part of a receptor complex with oncostatin M receptor (OSMR). |
| FBLN7 | Fibulin 7 | Predicted to enable calcium ion binding activity; heparan sulfate proteoglycan binding activity; and heparin binding activity. Predicted to be involved in cell adhesion. Predicted to act upstream of or within positive regulation of biomineralization. |
| FAM78A | Family with sequence similarity 78 member A | Broad expression in lymph node (RPKM 10.3), spleen (RPKM 9.5) and 20 other tissues. |
| PRMT7 | Protein arginine methyltransferase 7 | This gene encodes a member of the protein arginine N-methyltransferase family of proteins. The encoded enzyme transfers single methyl groups to arginine residues to generate monomethylarginines on histone proteins as well as other protein substrates. This enzyme plays a role in a wide range of biological processes, including neuronal differentiation, male germ line imprinting, small nuclear ribonucleoprotein biogenesis, and regulation of the Wnt signaling pathway. Mutations in this gene underlie multiple related syndromes in human patients characterized by intellectual disability, short stature and other features. The encoded protein may promote breast cancer cell invasion and metastasis in human patients. |
| PLA2G4C | Phospholipase A2 group IVC | This gene encodes a protein which is a member of the phospholipase A2 enzyme family which hydrolyzes glycerophospholipids to produce free fatty acids and lysophospholipids, both of which serve as precursors in the production of signaling molecules. The encoded protein has been shown to be a calcium-independent and membrane bound enzyme. |

Sources: National Institutes of Health National Library of Medicine, National Center for Biotechnology Information [https://www.ncbi.nlm.nih.gov/gene](https://www.ncbi.nlm.nih.gov/gene%20accessed%20August%2011)  National Human Genome Research Institute [https://www.genome.gov/genetics-glossary/Pseudogene](https://www.genome.gov/genetics-glossary/Pseudogene%20)  Last accessed August 16, 2024

**Supplementary Table 1B: Function of Top 50 mRNA differentially expressed between Cluster 1 and Cluster 2 subjects.**

| Gene Name | Gene Description | Gene type/ Function |
| --- | --- | --- |
| ATP2A3 | ATP2A3 (ATPase Sarcoplasmic/Endoplasmic Reticulum Ca2+ Transporting 3) also known as SERCA3 | This gene encodes one of the SERCA Ca(2+)-ATPases, which are intracellular pumps located in the sarcoplasmic or endoplasmic reticula of muscle cells. This enzyme catalyzes the hydrolysis of ATP coupled with the translocation of calcium from the cytosol to the sarcoplasmic reticulum lumen and participates in calcium sequestration associated with muscular excitation and contraction.  Diseases associated with ATP2A3 include Darier-White Disease and Atrophic Muscular Disease. Among its related pathways are Cardiac conduction and Pre-NOTCH Expression and Processing. Gene Ontology (GO) annotations related to this gene include nucleotide binding and P-type calcium transporter activity. An important paralog of this gene is ATP2A2. |
| PNCK | Pregnancy Up-Regulated Nonubiquitous CaM Kinase (Also known as BSTK3, CaMK1b) | Is a member of the calcium/calmodulin-dependent protein kinase family of protein serine/threonine kinases. Predicted to enable calmodulin binding activity and calmodulin-dependent protein kinase activity. Predicted to be involved in peptidyl-serine phosphorylation. Predicted to act upstream of or within protein phosphorylation. |
| CCDC88B | CCDC88B coiled-coil domain containing 88B [ Homo sapiens (human)] | This gene encodes a member of the hook-related protein family. Members of this family are characterized by an N-terminal potential microtubule binding domain, a central coiled-coiled and a C-terminal Hook-related domain. The encoded protein may be involved in linking organelles to microtubules. |
| CST4 | cystatin S | The cystatin superfamily encompasses proteins that contain multiple cystatin-like sequences. Some of the members are active cysteine protease inhibitors, while others have lost or perhaps never acquired this inhibitory activity. There are three inhibitory families in the superfamily, including the type 1 cystatins (stefins), type 2 cystatins and the kininogens. The type 2 cystatin proteins are a class of cysteine proteinase inhibitors found in a variety of human fluids and secretions. The cystatin locus on chromosome 20 contains the majority of the type 2 cystatin genes and pseudogenes. This gene is located in the cystatin locus and encodes a type 2 salivary cysteine peptidase inhibitor. The protein is an S-type cystatin, based on its elevated level of expression in saliva, tears and seminal plasma. The specific role in these fluids is unclear but antibacterial and antiviral activity is present, consistent with a protective function. |
| ARHGAP40 | Rho GTPase activating protein 40 | Predicted to enable GTPase activator activity. Predicted to be involved in regulation of actin filament polymerization and regulation of small GTPase mediated signal transduction. Predicted to be located in cytosol. Predicted to be active in cytoplasm. |
| CST1 | cystatin SN | The cystatin superfamily encompasses proteins that contain multiple cystatin-like sequences. Some of the members are active cysteine protease inhibitors, while others have lost or perhaps never acquired this inhibitory activity. There are three inhibitory families in the superfamily, including the type 1 cystatins (stefins), type 2 cystatins and the kininogens. The type 2 cystatin proteins are a class of cysteine proteinase inhibitors found in a variety of human fluids and secretions, where they appear to provide protective functions. The cystatin locus on chromosome 20 contains the majority of the type 2 cystatin genes and pseudogenes. This gene is located in the cystatin locus and encodes a cysteine proteinase inhibitor found in saliva, tears, urine, and seminal fluid. |
| LDLRAD2 | low density lipoprotein receptor class A domain containing 2 | Predicted to be integral component of membrane. |
| PRB1 | proline rich protein BstNI subfamily 1 | Predicted to be located in extracellular region. Genetic polymorphisms result in both protein coding and non-coding alleles of this gene. |
| CCDC183 | coiled-coil domain containing 183 | Function not fully elucidated yet |
| RILP | Rab interacting lysosomal protein | This gene encodes a lysosomal protein that interacts with RAB7, a small GTPase that controls transport to endocytic degradative compartments. Studies using mutant forms of the two proteins suggest that this protein represents a downstream effector for RAB7, and both proteins act together in the regulation of late endocytic traffic. A unique region of this protein has also been shown to be involved in the regulation of lysosomal morphology. |
| MAPK8IP3 | mitogen-activated protein kinase 8 interacting protein 3 | The protein encoded by this gene shares similarity with the product of Drosophila syd gene, required for the functional interaction of kinesin I with axonal cargo. Studies of the similar gene in mouse suggested that this protein may interact with and regulate the activity of numerous protein kinases of the JNK signaling pathway, and thus function as a scaffold protein in neuronal cells. The C. elegans counterpart of this gene is found to regulate synaptic vesicle transport possibly by integrating JNK signaling, and kinesin-1 transport. |
| PLXNB3 | plexin B3 | The protein encoded by this gene is a member of the plexin family. It functions as a receptor for semaphorin 5A, and plays a role in axon guidance, invasive growth and cell migration. |
| SRPK3 | SRSF protein kinase 3 | This gene encodes a protein kinase similar to a protein kinase which is specific for the SR (serine/arginine-rich domain) family of splicing factors. A highly similar protein has been shown to play a role in muscle development in mice. |
| ARHGF16 | Rho guanine nucleotide exchange factor 16 | Although the specific function of this protein is not known yet, it is thought to be involved in protein-protein and protein-lipid interactions. |
| CARD14 | caspase recruitment domain family member 14 | This gene encodes a caspase recruitment domain-containing protein that is a member of the membrane-associated guanylate kinase (MAGUK) family of proteins. Members of this protein family are scaffold proteins that participate in a diverse array of cellular processes including cellular adhesion, signal transduction and cell polarity control. This protein has been shown to specifically interact with BCL10, a protein known to function as a positive regulator of cell apoptosis and NF-kappaB activation. |
| CHDH | choline dehydrogenase | The protein encoded by this gene is a choline dehydrogenase that localizes to the mitochondrion. Variations in this gene can affect susceptibility to choline deficiency. A few transcript variants have been found for this gene, but the full-length nature of only one has been characterized to date. |
| JSRP1 | junctional sarcoplasmic reticulum protein 1 | The protein encoded by this gene participates in excitation-contraction coupling at the sarcoplasmic reticulum. The encoded protein can interact with CACNA1S, CACNB1, and calsequestrin to help regulate calcium influx and efflux in skeletal muscle. |
| PTPRH | protein tyrosine phosphatase receptor type H | The protein encoded by this gene is a member of the protein tyrosine phosphatase (PTP) family. PTPs are known to be signaling molecules that regulate a variety of cellular processes including cell growth, differentiation, mitotic cycle, and oncogenic transformation. This PTP possesses an extracellular region, a single transmembrane region, and a single intracytoplasmic catalytic domain, and thus represents a receptor-type PTP. The extracellular region contains eight fibronectin type III-like repeats and multiple N-glycosylation sites. The gene was shown to be expressed primarily in brain and liver, and at a lower level in heart and stomach. It was also found to be expressed in several cancer cell lines, but not in the corresponding normal tissues. |
| ATP10B | ATPase phospholipid transporting 10B | Enables glycosylceramide flippase activity and phosphatidylcholine flippase activity. Involved in lysosomal membrane organization. Located in endoplasmic reticulum. Is integral component of lysosomal membrane. Part of phospholipid-translocating ATPase complex. |
| FUT3 | Also known as CD174  fucosyltransferase 3 (Lewis blood group) | The Lewis histo-blood group system comprises a set of fucosylated glycosphingolipids that are synthesized by exocrine epithelial cells and circulate in body fluids. The glycosphingolipids function in embryogenesis, tissue differentiation, tumor metastasis, inflammation, and bacterial adhesion. They are secondarily absorbed to red blood cells giving rise to their Lewis phenotype. This gene is a member of the fucosyltransferase family, which catalyzes the addition of fucose to precursor polysaccharides in the last step of Lewis antigen biosynthesis. It encodes an enzyme with alpha(1,3)-fucosyltransferase and alpha(1,4)-fucosyltransferase activities. Mutations in this gene are responsible for the majority of Lewis antigen-negative phenotypes. Differences in the expression of this gene are associated with host susceptibility to viral infection. |
| EGLN3 | egl-9 family hypoxia inducible factor 3 | Enables peptidyl-proline 4-dioxygenase activity. Involved in several processes, including activation of cysteine-type endopeptidase activity involved in apoptotic process; peptidyl-proline hydroxylation to 4-hydroxy-L-proline; and response to hypoxia. Located in cytosol and nucleus. Implicated in renal cell carcinoma. Biomarker of clear cell renal cell carcinoma. |
| TMEM200A | transmembrane protein 200A | Predicted to be integral component of membrane. |
| HBA2 | hemoglobin subunit alpha 2 | The human alpha globin gene cluster located on chromosome 16 spans about 30 kb and includes seven loci: 5'- zeta - pseudozeta - mu - pseudoalpha-1 - alpha-2 - alpha-1 - theta - 3'. The alpha-2 (HBA2) and alpha-1 (HBA1) coding sequences are identical. These genes differ slightly over the 5' untranslated regions and the introns, but they differ significantly over the 3' untranslated regions. Two alpha chains plus two beta chains constitute HbA, which in normal adult life comprises about 97% of the total hemoglobin; alpha chains combine with delta chains to constitute HbA-2, which with HbF (fetal hemoglobin) makes up the remaining 3% of adult hemoglobin. Alpha thalassemias result from deletions of each of the alpha genes as well as deletions of both HBA2 and HBA1; some nondeletion alpha thalassemias have also been reported. |
| PRLR | prolactin receptor | This gene encodes a receptor for the anterior pituitary hormone, prolactin, and belongs to the type I cytokine receptor family. Prolactin-dependent signaling occurs as the result of ligand-induced dimerization of the prolactin receptor. Several alternatively spliced transcript variants encoding different membrane-bound and soluble isoforms have been described for this gene, which may function to modulate the endocrine and autocrine effects of prolactin in normal tissue and cancer. |
| LRFN5 | leucine rich repeat and fibronectin type III domain containing 5 | Codes a protein that belongs to the leucine-rich repeat and fibronectin type III domain-containing family of proteins. A similar protein in mouse, a glycosylated transmembrane protein, is thought to function in presynaptic differentiation. |
| FRMD6 | FERM domain containing 6 | Predicted to be involved in actomyosin structure organization. Predicted to act upstream of or within apical constriction; cellular protein localization; and regulation of actin filament-based process. Predicted to be located in apical junction complex. Predicted to be active in cytoskeleton. |
| LUM | lumican | Protein-coding  This gene encodes a member of the small leucine-rich proteoglycan (SLRP) family that includes decorin, biglycan, fibromodulin, keratocan, epiphycan, and osteoglycin. In these bifunctional molecules, the protein moiety binds collagen fibrils and the highly charged hydrophilic glycosaminoglycans regulate interfibrillar spacings. Lumican is the major keratan sulfate proteoglycan of the cornea but is also distributed in interstitial collagenous matrices throughout the body. Lumican may regulate collagen fibril organization and circumferential growth, corneal transparency, and epithelial cell migration and tissue repair. |
| JAM2 | junctional adhesion molecule 2 | This gene belongs to the immunoglobulin superfamily, and the junctional adhesion molecule (JAM) family. The protein encoded by this gene is a type I membrane protein that is localized at the tight junctions of both epithelial and endothelial cells. It acts as an adhesive ligand for interacting with a variety of immune cell types and may play a role in lymphocyte homing to secondary lymphoid organs. |
| CNTN1 | Contactin 1 | The protein encoded by this gene is a member of the immunoglobulin superfamily. It is a glycosylphosphatidylinositol (GPI)-anchored neuronal membrane protein that functions as a cell adhesion molecule. It may play a role in the formation of axon connections in the developing nervous system. |
| SNHG14 | Small nucleolar RNA host gene 14 | Non-coding RNA. |
| STAC | SH3 and cysteine rich domain | Predicted to enable transmembrane transporter binding activity. Predicted to be involved in positive regulation of protein localization to plasma membrane; positive regulation of voltage-gated calcium channel activity; and skeletal muscle contraction. Predicted to act upstream of or within cellular response to heat; muscle contraction; and regulation of voltage-gated calcium channel activity. Predicted to be located in T-tubule. Predicted to be extrinsic component of cytoplasmic side of plasma membrane |
| MCF2 | MCF.2 cell line derived transforming sequence | The oncogenic protein encoded by this gene is a guanine nucleotide exchange factor (GEF) that exerts control over some members of the Rho family of small GTPases. Several transcript variants encoding different isoforms have been found for this gene. These isoforms exhibit different expression patterns and varying levels of GEF activity. |
| WNT5A | Wnt family member 5A | The WNT gene family consists of structurally related genes which encode secreted signaling proteins. These proteins have been implicated in oncogenesis and in several developmental processes, including regulation of cell fate and patterning during embryogenesis. This gene encodes a member of the WNT family that signals through both the standard and non-canonical WNT pathways. This protein is a ligand for the seven transmembrane receptor frizzled-5 and the tyrosine kinase orphan receptor 2. This protein plays an essential role in regulating developmental pathways during embryogenesis. This protein may also play a role in oncogenesis. Mutations in this gene are the cause of autosomal dominant Robinow syndrome. |
| NELL2 | Neural EGFL like 2 | The protein encoded by this gene is a glycoprotein containing several von Willebrand factor C domains and epidermal growth factor (EGF)-like domains. The encoded protein acts as a homotrimer and is found in the cytoplasm. Several variants encoding a few different isoforms exist, and at least one isoform appears to be a secreted protein. Studies in mouse suggest that this protein plays a role in neural cell growth and differentiation as well as in oncogenesis. |
| HMGN1P36 | High mobility group nucleosome binding domain 1 pseudogene 36 | Pseudogene; A pseudogene is a segment of DNA that structurally resembles a gene but is not capable of coding for a protein. Pseudogenes are most often derived from genes that have lost their protein-coding ability due to accumulated mutations that have occurred over the course of evolution. |
| PTN | Pleiotrophin | The protein encoded by this gene is a secreted heparin-binding growth factor. The protein has significant roles in cell growth and survival, cell migration, angiogenesis and tumorigenesis. Alternative splicing and the use of alternative promoters results in multiple transcript variants. |
| SCN2B | Sodium voltage-gated channel beta subunit 2 | The protein encoded by this gene is the beta 2 subunit of the type II voltage-gated sodium channel. The encoded protein participates in cell-cell adhesion and cell migration. Defects in this gene can be a cause of Brugada Syndrome, atrial fibrillation, or sudden infant death syndrome. |
| AR | Androgen receptor | The androgen receptor gene is more than 90 kb long and codes for a protein that has 3 major functional domains: the N-terminal domain, DNA-binding domain, and androgen-binding domain. The protein functions as a steroid-hormone activated transcription factor. Upon binding the hormone ligand, the receptor dissociates from accessory proteins, translocates into the nucleus, dimerizes, and then stimulates transcription of androgen responsive genes. This gene contains 2 polymorphic trinucleotide repeat segments that encode polyglutamine and polyglycine tracts in the N-terminal transactivation domain of its protein. Expansion of the polyglutamine tract from the normal 9-34 repeats to the pathogenic 38-62 repeats causes spinal bulbar muscular atrophy (SBMA, also known as Kennedy's disease). Mutations in this gene are also associated with complete androgen insensitivity (CAIS). Alternative splicing results in multiple transcript variants encoding different isoforms. |
| SNORD116-18 | Small Nucleolar RNA, C/D Box 116-18 | *snoRNA |
| SNORD 116-25 | Small Nucleolar RNA, C/D Box 116-25 | *snoRNA |
| TCP1 | T-complex 1 | The protein encoded by this gene is a molecular chaperone that is a member of the chaperonin containing TCP1 complex (CCT), also known as the TCP1 ring complex (TRiC). This complex consists of two identical stacked rings, each containing eight different proteins. Unfolded polypeptides enter the central cavity of the complex and are folded in an ATP-dependent manner. The complex folds various proteins, including actin and tubulin. Alternate transcriptional splice variants of this gene, encoding different isoforms, have been characterized. In addition, three pseudogenes that appear to be derived from this gene have been found. |
| SNORD62A | Small nucleolar RNA, C/D box 62A | *snoRNA |
| SNORD62B | Small nucleolar RNA, C/D box 62B | *snoRNA |
| GPC3 | Glypican 3 | Cell surface heparan sulfate proteoglycans are composed of a membrane-associated protein core substituted with a variable number of heparan sulfate chains. Members of the glypican-related integral membrane proteoglycan family (GRIPS) contain a core protein anchored to the cytoplasmic membrane via a glycosyl phosphatidylinositol linkage. These proteins may play a role in the control of cell division and growth regulation. The protein encoded by this gene can bind to and inhibit the dipeptidyl peptidase activity of CD26, and it can induce apoptosis in certain cell types. |
| CCDC36 | Coiled-coil domain containing 36 | Protein-coding |
| CSMD3 | CUB and Sushi multiple domains 3 | Predicted to be involved in regulation of dendrite development. Predicted to be located in plasma membrane. |
| SLIT2 | Slit guidance ligand 2 | This gene encodes a member of the slit family of secreted glycoproteins, which are ligands for the Robo family of immunoglobulin receptors. Slit proteins play highly conserved roles in axon guidance and neuronal migration and may also have functions during other cell migration processes including leukocyte migration. Members of the slit family are characterized by an N-terminal signal peptide, four leucine-rich repeats, nine epidermal growth factor repeats, and a C-terminal cysteine knot. Proteolytic processing of this protein gives rise to an N-terminal fragment that contains the four leucine-rich repeats, and five epidermal growth factor repeats and a C-terminal fragment that contains four epidermal growth factor repeats and the cysteine knot. Both full length and cleaved proteins are secreted extracellularly and can function in axon repulsion as well as other specific processes. |
| ZNF660 | Zinc finger protein 660 | This gene encodes a protein that contains multiple C2H2 zinc finger domains and is located in a cluster of zinc-finger encoding genes on chromosome 3. Naturally-occurring readthrough transcription is observed between this gene and the downstream zinc finger protein 197 gene |
| DPP6 | Dipeptidyl peptidase like 6 | This gene encodes a single-pass type II membrane protein that is a member of the peptidase S9B family of serine proteases. This protein has no detectable protease activity, most likely due to the absence of the conserved serine residue normally present in the catalytic domain of serine proteases. However, it does bind specific voltage-gated potassium channels and alters their expression and biophysical properties. Variations in this gene may be associated with susceptibility to amyotrophic lateral sclerosis and with idiopathic ventricular fibrillation. |
| NPNT | Nephronectin | Predicted to enable integrin binding activity. Predicted to be involved in several processes, including cell-cell adhesion mediated by integrin; positive regulation of ERK1 and ERK2 cascade; and positive regulation of osteoblast differentiation. Predicted to act upstream of or within positive regulation of transforming growth factor beta receptor signaling pathway. Located in extracellular exosome. Part of collagen-containing extracellular matrix. |

Sources: National Institutes of Health National Library of Medicine, National Center for Biotechnology Information [https://www.ncbi.nlm.nih.gov/gene](https://www.ncbi.nlm.nih.gov/gene%20accessed%20August%2011)  National Human Genome Research Institute [https://www.genome.gov/genetics-glossary/Pseudogene](https://www.genome.gov/genetics-glossary/Pseudogene%20)  Last accessed August 16, 2024

- 1. **SUPPLEMENTARY REFERENCES**

(A1) Liu C, Du H, Wang Y, et al. S100A11 regulates nasal epithelial cell remodeling and inflammation in CRSwNPs via the RAGE-mediated AMPK-STAT3 pathway. *Mol Immunol*. 2021;140:35-46.

(A2) Lacy P. The role of Rho GTPases and SNAREs in mediator release from granulocytes. *Pharmacol Ther*. 2005;107(3):358-376.

(A3) Blander JM, Medzhitov R. Regulation of phagosome maturation by signals from toll-like receptors. *Science*. 2004;304(5673):1014-1018.

(A4) Wang M, Tang S, Yang X, et al. Identification of key genes and pathways in chronic rhinosinusitis with nasal polyps and asthma comorbidity using bioinformatics approaches. *Front Immunol*. 2022;13:941547. Published 2022 Aug 17.

(A5) Huoman J, Sayyab S, Apostolou E, et al. Epigenetic rewiring of pathways related to odour perception in immune cells exposed to SARS-CoV-2 *in vivo* and *in vitro*. *Epigenetics*. 2022;17(13):1875-1891.

(A6) Sanchez-Lopez E, Zhong Z, Stubelius A, et al. Choline Uptake and Metabolism Modulate Macrophage IL-1β and IL-18 Production. *Cell Metab*. 2019;29(6):1350-1362.e7.

(A7) Michaeloudes C, Abubakar-Waziri H, Lakhdar R, et al. Molecular mechanisms of oxidative stress in asthma. *Mol Aspects Med*. 2022;85:101026.

(A8) Nakamura M, Sadoshima J. Mechanisms of physiological and pathological cardiac hypertrophy. *Nat Rev Cardiol*. 2018;15(7):387-407.

(A9) Behrendt AK, Hansen G. CD27 costimulation is not critical for the development of asthma and respiratory tolerance in a murine model. *Immunol Lett*. 2010;133(1):19-27.

(A10) Germann M, Gallo C, Donahue T, et al. Characterizing sterol defect suppressors uncovers a novel transcriptional signaling pathway regulating zymosterol biosynthesis. *J Biol Chem*. 2005;280(43):35904-35913.

(A11) Cardoso D, Perucha E. Cholesterol metabolism: a new molecular switch to control inflammation. *Clin Sci (Lond)*. 2021;135(11):1389-1408.

(A12) Wang M, Tang S, Yang X, et al. Identification of key genes and pathways in chronic rhinosinusitis with nasal polyps and asthma comorbidity using bioinformatics approaches. *Front Immunol*. 2022;13:941547. Published 2022 Aug 17.

(A13) Wang H, Xu J, Lazarovici P, Quirion R, Zheng W. cAMP Response Element-Binding Protein (CREB): A Possible Signaling Molecule Link in the Pathophysiology of Schizophrenia. *Front Mol Neurosci*. 2018;11:255. Published 2018 Aug 30.

(A14) Wang T, Liu X, Hao M, et al. Design, synthesis and evaluation of pyrrolo[2,3-d]pyrimidine-phenylamide hybrids as potent Janus kinase 2 inhibitors. *Bioorg Med Chem Lett*. 2016;26(12):2936-2941.

(A15) Wei F, Gao C, Wang Y. The role of influenza A virus-induced hypercytokinemia. *Crit Rev Microbiol*. 2022;48(2):240-256.

(A16) Duerr CU, McCarthy CD, Mindt BC, et al. Type I interferon restricts type 2 immunopathology through the regulation of group 2 innate lymphoid cells. *Nat Immunol*. 2016;17(1):65-75.(A17) Chan YS, Yang L, Ng HH. Transcriptional regulatory networks in embryonic stem cells. *Prog Drug Res*. 2011;67:239-252.

(A18) Dipankar P, Kumar P, Dash SP, Sarangi PP. Functional and Therapeutic Relevance of Rho GTPases in Innate Immune Cell Migration and Function during Inflammation: An *In Silico* Perspective. *Mediators Inflamm*. 2021;2021:6655412.

(A19) Haan C, Rolvering C, Raulf F, et al. Jak1 has a dominant role over Jak3 in signal transduction through γc-containing cytokine receptors. *Chem Biol*. 2011;18(3):314-323.

(A20) Bros M, Haas K, Moll L, Grabbe S. RhoA as a Key Regulator of Innate and Adaptive Immunity. *Cells*. 2019;8(7):733.

(A21) Gaesser JM, Fyffe-Maricich SL. Intracellular signaling pathway regulation of myelination and remyelination in the CNS. *Exp Neurol*. 2016;283(Pt B):501-511.

(A22) Chen CY, Cheng YY, Yen CY, Hsieh PC. Mechanisms of pluripotency maintenance in mouse embryonic stem cells. *Cell Mol Life Sci*. 2017;74(10):1805-1817.

(A23) Mossahebi-Mohammadi M, Quan M, Zhang JS, Li X. FGF Signaling Pathway: A Key Regulator of Stem Cell Pluripotency. *Front Cell Dev Biol*. 2020;8:79.

(A24) Moradi F, Babashah S, Sadeghizadeh M, Jalili A, Hajifathali A, Roshandel H. Signaling pathways involved in chronic myeloid leukemia pathogenesis: The importance of targeting Musashi2-Numb signaling to eradicate leukemia stem cells. *Iran J Basic Med Sci*. 2019;22(6):581-589.

(A25) Yu J, Liu D, Sun X, et al. CDX2 inhibits the proliferation and tumor formation of colon cancer cells by suppressing Wnt/β-catenin signaling via transactivation of GSK-3β and Axin2 expression. *Cell Death Dis*. 2019;10(1):26.

(A26) Wu D, Mueller SK, Nocera AL, Finn K, Libermann TA, Bleier BS. Axonal Guidance Signaling Pathway Is Suppressed in Human Nasal Polyps. *Am J Rhinol Allergy*. 2018;32(4):208-216.

(A27) Wang T, Liu X, Hao M, et al. Design, synthesis and evaluation of pyrrolo[2,3-d]pyrimidine-phenylamide hybrids as potent Janus kinase 2 inhibitors. *Bioorg Med Chem Lett*. 2016;26(12):2936-2941.

(A28) Yao Q, Wu X, Tao C, et al. Osteoarthritis: pathogenic signaling pathways and therapeutic targets. *Signal Transduct Target Ther*. 2023;8(1):56.

(A29) Gaesser JM, Fyffe-Maricich SL. Intracellular signaling pathway regulation of myelination and remyelination in the CNS. *Exp Neurol*. 2016;283(Pt B):501-511.

(A30) Mossahebi-Mohammadi M, Quan M, Zhang JS, Li X. FGF Signaling Pathway: A Key Regulator of Stem Cell Pluripotency. *Front Cell Dev Biol*. 2020;8:79.

(A31) Meng F, Wang K, Aoyama T, et al. Interleukin-17 signaling in inflammatory, Kupffer cells, and hepatic stellate cells exacerbates liver fibrosis in mice. *Gastroenterology*. 2012;143(3):765-776.e3.

(A32) Wang M, Tang S, Yang X, et al. Identification of key genes and pathways in chronic rhinosinusitis with nasal polyps and asthma comorbidity using bioinformatics approaches. *Front Immunol*. 2022;13:941547.

(A33) Nakamura M, Sadoshima J. Mechanisms of physiological and pathological cardiac hypertrophy. *Nat Rev Cardiol*. 2018;15(7):387-407.

(A34) Wang H, Xu J, Lazarovici P, Quirion R, Zheng W. cAMP Response Element-Binding Protein (CREB): A Possible Signaling Molecule Link in the Pathophysiology of Schizophrenia. *Front Mol Neurosci*. 2018;11:255.

(A35) Bose S, Purkait D, Joseph D, Nayak V, Subramanian R. Structural and functional characterization of CMP-N-acetylneuraminate synthetase from Vibrio cholerae. *Acta Crystallogr D Struct Biol*. 2019;75(Pt 6):564-577.

(A36) González F. Inflammation in Polycystic Ovary Syndrome: underpinning of insulin resistance and ovarian dysfunction. *Steroids*. 2012;77(4):300-305.

(A37) Wu D, Mueller SK, Nocera AL, Finn K, Libermann TA, Bleier BS. TREM-1 Neutrophil Activation Pathway Is Suppressed in Eosinophilic Nasal Polyps. *Am J Rhinol Allergy*. 2018;32(5):359-368.

(A38) Cardoso D, Perucha E. Cholesterol metabolism: a new molecular switch to control inflammation. *Clin Sci (Lond)*. 2021;135(11):1389-1408.

(A39) Ross EA, Devitt A, Johnson JR. Macrophages: The Good, the Bad, and the Gluttony. *Front Immunol*. 2021;12:708186. Published 2021 Aug 12.

(A40) Kim JA, Kim S, Kim JE, et al. Activation of the Intrinsic Coagulation Pathway in Patients With Chronic Urticaria. *Allergy Asthma Immunol Res*. 2015;7(5):476-482.

(A41) Laidlaw TM, Mullol J, Woessner KM, Amin N, Mannent LP. Chronic Rhinosinusitis with Nasal Polyps and Asthma. *J Allergy Clin Immunol Pract*. 2021;9(3):1133-1141.

(A42) Jere SW, Abrahamse H, Houreld NN. The JAK/STAT signaling pathway and photobiomodulation in chronic wound healing. *Cytokine Growth Factor Rev*. 2017;38:73-79.

(A43) Hu L, Liu R, Zhang L. Advance in bone destruction participated by JAK/STAT in rheumatoid arthritis and therapeutic effect of JAK/STAT inhibitors. *Int Immunopharmacol*. 2022;111:109095.

(A44) Blander JM, Medzhitov R. Regulation of phagosome maturation by signals from toll-like receptors. *Science*. 2004;304(5673):1014-1018.

(A45) Young MJ. Mechanisms of mineralocorticoid receptor-mediated cardiac fibrosis and vascular inflammation. *Curr Opin Nephrol Hypertens*. 2008;17(2):174-180.

(A46) Into T, Horie T, Inomata M, et al. Basal autophagy prevents autoactivation or enhancement of inflammatory signals by targeting monomeric MyD88. *Sci Rep*. 2017;7(1):1009.

(A47) Schmidt SI, Blaabjerg M, Freude K, Meyer M. RhoA Signaling in Neurodegenerative Diseases. *Cells*. 2022;11(9):1520. Published 2022 May 1.

(A48) Meng F, Wang K, Aoyama T, et al. Interleukin-17 signaling in inflammatory, Kupffer cells, and hepatic stellate cells exacerbates liver fibrosis in mice. *Gastroenterology*. 2012;143(3):765-776.e3.

(A49) Li Y, Chang LH, Huang WQ, et al. IL-17A mediates pyroptosis via the ERK pathway and contributes to steroid resistance in CRSwNP. *J Allergy Clin Immunol*. 2022;150(2):337-351.

(A50) Ju Lee H, Bartsch D, Xiao C, et al. A post-transcriptional program coordinated by CSDE1 prevents intrinsic neural differentiation of human embryonic stem cells. *Nat Commun*. 2017;8(1):1456. Published 2017 Nov 13.

(A51) Turowski TW, Boguta M. Specific Features of RNA Polymerases I and III: Structure and Assembly. *Front Mol Biosci*. 2021;8:680090.

(A52) Jere SW, Abrahamse H, Houreld NN. The JAK/STAT signaling pathway and photobiomodulation in chronic wound healing. *Cytokine Growth Factor Rev*. 2017;38:73-79.

(A53) Hu L, Liu R, Zhang L. Advance in bone destruction participated by JAK/STAT in rheumatoid arthritis and therapeutic effect of JAK/STAT inhibitors. *Int Immunopharmacol*. 2022;111:109095.

(A54) Epstein Shochet G, Brook E, Bardenstein-Wald B, Shitrit D. TGF-β pathway activation by idiopathic pulmonary fibrosis (IPF) fibroblast derived soluble factors is mediated by IL-6 trans-signaling. *Respir Res*. 2020;21(1):56.

(A55) Kobayashi Y, Iwata A, Suzuki K, et al. B and T lymphocyte attenuator inhibits LPS-induced endotoxic shock by suppressing Toll-like receptor 4 signaling in innate immune cells [published correction appears in Proc Natl Acad Sci U S A. 2018 Apr 9;]. *Proc Natl Acad Sci U S A*. 2013;110(13):5121-5126.

(A56) Papayannopoulos V. Neutrophil extracellular traps in immunity and disease. *Nat Rev Immunol*. 2018;18(2):134-147.

(A57) Into T, Horie T, Inomata M, et al. Basal autophagy prevents autoactivation or enhancement of inflammatory signals by targeting monomeric MyD88. *Sci Rep*. 2017;7(1):1009.

(A58) Lu Y, Basatemur G, Scott IC, et al. Interleukin-33 Signaling Controls the Development of Iron-Recycling Macrophages. *Immunity*. 2020;52(5):782-793.e5.

(A59) Meng F, Wang K, Aoyama T, et al. Interleukin-17 signaling in inflammatory, Kupffer cells, and hepatic stellate cells exacerbates liver fibrosis in mice. *Gastroenterology*. 2012;143(3):765-776.e3.

(A60) Nurden AT. Clinical significance of altered collagen-receptor functioning in platelets with emphasis on glycoprotein VI. *Blood Rev*. 2019;38:100592.
